# Supplementary material for: Measuring statistics-induced entanglement entropy with a Hong–Ou–Mandel interferometer
Source: Nat Commun. 2024 Apr 23;15:3428. doi: 10.1038/s41467-024-47335-z (PMC11039745; doi:10.1038/s41467-024-47335-z)
Supplement: Supplementary file 1 — Supplementary Infomation [file 41467_2024_47335_MOESM1_ESM.pdf]

Supplementary Information for "Measuring statistics-induced entanglement entropy with a Hong-Ou-Mandel interferometer"

Gu Zhang, Changki Hong, Tomer Alkalay, Vladimir Umansky, Moty Heiblum, Igor Gornyi, and Yuval Gefen

(Dated: March 12, 2024)

In this Supplementary Information, we provide details on: (i) Deriving entanglement entropy from full counting statistics, (ii) Influence of Coulomb interactions on statistics quantification functions, (iii) Statistics-induced entanglement in terms of Bell pairs, (iv) The dwell time and the measuring time, (v) Additional experimental data on $\nu = 3$ and $\nu = 1$ measurements, (vi) Possible SEE application: quantifying entanglement of a mixed state, and (vii) EE and SEE with a finite scattering area.

S1. DERIVING ENTANGLEMENT ENTROPY FROM FULL COUNTING STATISTICS

S1A. Generating function of full counting statistics

In this section, we express the FCS-generating function of a free-particle HOM interferometer in terms of the spectrum function $\mu(z)$ of the correlation matrix M after scatterings [see its definition after (S4)]. Before scatterings, the system state is described by the diagonal zero-temperature correlation matrix n_0 with elements $\langle \epsilon, \alpha | n_0 | \epsilon', \alpha' \rangle = \Theta(V_\alpha - \epsilon)\delta_{\alpha,\alpha'}\delta(\epsilon - \epsilon')$ labeled by the energy ϵ and arm α indices, with $\Theta(\epsilon)$ the step function. Here $V_\alpha = V_{\text{bias}}$ for two sources $\alpha = \mathcal{S}_A, \mathcal{S}_B$ and is zero for two grounded middle arms $\alpha = \mathcal{M}_A, \mathcal{M}_B$.

After scatterings, following Ref. [S1], we write down the general expression of the generating function

$$\chi(\lambda_{\text{FCS}}) = \det(1 - n + n U_{\mathcal{T}}^\dagger e^{i\lambda_{\text{FCS}} P_A} U_{\mathcal{T}} e^{-i\lambda_{\text{FCS}} P_A}), \tag{S1}$$

where $U_{\mathcal{T}}$ refers to the scattering matrix of the central QPC, λ_{FCS} is the measuring field in FCS, P_A is the projection operator onto the subsystem A, and $n = U_0 n_0 U_0^\dagger$ is the original correlation matrix n_0, after the transformation

$$U_0 = \begin{pmatrix} r_A & t_A & 0 & 0 \\ -t_A^* & r_A^* & 0 & 0 \\ 0 & 0 & r_B & t_B \\ 0 & 0 & -t_B^* & r_B^* \end{pmatrix} \tag{S2}$$

that represents scatterings at two diluters. It acts on a four-dimensional spinor with indexes respectively referring to the upper source $\mathcal{S}_A$, two middle arms $\mathcal{M}_A$, $\mathcal{M}_B$, and the lower source $\mathcal{S}_B$. In Eq. (S2), the matrix elements $|t_A|^2 = \mathcal{T}_A$ and $|t_B|^2 = \mathcal{T}_B$ for transmission and $|r_A|^2 = 1 - \mathcal{T}_A$ and $|r_B|^2 = 1 - \mathcal{T}_B$ for reflections. To proceed, we

rewrite Eq. (S1) as

$$\begin{aligned}\chi(\lambda_{\text{FCS}}) &= \det\left(U_0U_0^\dagger - U_0n_0U_0^\dagger + U_0n_0U_0^\dagger e^{-i\lambda_{\text{FCS}}P_A}U_0U_0^\dagger U^\dagger e^{i\lambda_{\text{FCS}}P_A}U(U_0U_0^\dagger)\right)\\ &= \det\left[1-n_0+n_0\left(U_0^\dagger e^{-i\lambda_{\text{FCS}}P_A}U_0\right)\left(U_c^\dagger e^{i\lambda_{\text{FCS}}P_A}U_c\right)\right]\\ &= \det\left[1-n_0+n_0e^{-i\lambda_{\text{FCS}}P_A}\left(U_c^\dagger e^{i\lambda_{\text{FCS}}P_A}U_c\right)\right],\end{aligned} \tag{S3}$$

where $U_c = U_{\mathcal{T}}U_0$. In the first line of Eq. (S3), we simply inserted identities $U_0U_0^\dagger = 1$. In the second line, we have used the fact that $\det(m_1m_2m_3) = \det(m_1)\det(m_2)\det(m_3)$ for square matrices, and that $\det(U_0) = 1$. Finally, in the last line, we have used the fact that P_A commutes with U_0. The derivation that follows is quite similar to that of Ref. [S1]:

$$\begin{aligned}\chi(\lambda_{\text{FCS}}) &= \det\left\{e^{i\lambda_{\text{FCS}}P_An_0}\left[1-n_0+n_0e^{-i\lambda_{\text{FCS}}P_A}\left(U_c^\dagger e^{i\lambda_{\text{FCS}}P_A}U_c\right)\right]e^{-i\lambda_{\text{FCS}}P_An_0}\right\}\\ &= \det\left[\left(1-n_U+n_Ue^{i\lambda_{\text{FCS}}P_A}\right)U_ce^{-i\lambda_{\text{FCS}}P_An_0}U_c^\dagger\right]\\ &= \det\left[\left(1+P_An_U(e^{i\lambda_{\text{FCS}}}-1)P_A\right)U_ce^{-i\lambda_{\text{FCS}}P_An_0}U_c^\dagger\right]\\ &= \det\left[\left(1+M(e^{i\lambda_{\text{FCS}}}-1)\right)U_ce^{-i\lambda_{\text{FCS}}P_An_0}U_c^\dagger\right],\end{aligned} \tag{S4}$$

where $n_U = U_cn_0U_c^\dagger$ and $M = P_An_UP_A$. After the substitution $z \equiv (1-e^{i\lambda_{\text{FCS}}})$, we arrive at the expression

$$\chi(z) = \det\left[(z-M)\frac{1}{z}e^{-i\lambda_{\text{FCS}}(z)U_cP_An_0U_c^\dagger}\right]. \tag{S5}$$

Notice that if we add a small imaginary part to z, the spectral function of M can be found as

$$\mu(z) = \frac{1}{\pi}\text{Im}\partial_z\left[\ln\chi(z-i\eta)\right] + \mathcal{C}_1\delta(z) + \mathcal{C}_2\delta(z-1), \tag{S6}$$

where $\eta > 0$ is an infinitesimal, and $\mathcal{C}_1$, $\mathcal{C}_2$ are two constant numbers. The term $\mathcal{C}_1\delta(z)$ originates from the $1/z$ term of Eq. (S5), and that of $\mathcal{C}_2$ comes from the exponential phase factor.

Equation (S6) almost coincides with the expression for the generating function in Refs. [S1, S2] obtained for a single-QPC model, except for the values of $\mathcal{C}_1$ and $\mathcal{C}_2$. However, these two constant numbers will not enter the integral over the spectral function, and are thus irrelevant to the EE. We also emphasize that Eq. (S6) is valid for any finite number of co-propagating channels bridged by finite numbers of independent scattering QPCs, given the measuring time is much longer than the traveling time between any two QPCs.

S1B. The EE evaluated from the spectral function of the correlation matrix

In S1A, we have shown the connection between the FCS generating function $\chi(z)$ and the spectrum function $\mu(z)$ of the correlation function matrix M. In this section, we relate $\mu(z)$ to the EE, through which the EE-FCS connection can be established.

We consider a general case where a non-interacting subsystem is described by the reduced density matrix ρ_{sub}

$$\rho_{\text{sub}} = \frac{1}{Z} \exp\left(-\tilde{H}_{ij} a_i^\dagger a_j\right), \tag{S7}$$

with the matrix elements $\tilde{H}_{ij}$. The operators $a_i^\dagger$ and $a_j^\dagger$ act on states that belong to the subsystem we consider. In Eq. (S7), the factor Z

$$Z = \det\left(1 + e^{-\tilde{H}}\right) \tag{S8}$$

is $\tilde{H}$-dependent, and is introduced due to the normalization requirement $\text{Tr}(\rho_{\text{sub}}) = 1$.

Following Eq. (S7), we define an m matrix whose element

$$m_{ij} = \text{Tr}(\rho_{\text{sub}} a_i^\dagger a_j) = \left[(e^{\tilde{H}} + 1)^{-1}\right]_{ij} \tag{S9}$$

is the correlation function of particles in states i and j. Now, the entanglement entropy equals

$$\begin{aligned} S_{\text{ent}} &= -\text{Tr}(\rho_{\text{sub}} \ln \rho_{\text{sub}}) = -\text{Tr}\left[\rho_{\text{sub}}\left(-\sum_{ij} \tilde{H}_{ij} a_i^\dagger a_j\right)\right] + \text{Tr}\left[\rho_{\text{sub}} \ln Z\right] \\ &= \sum_{ij} \tilde{H}_{ij} m_{ij} + \ln\left[\det\left(1 + e^{-\tilde{H}}\right)\right] \\ &= \text{Tr}(\tilde{H} m) + \text{Tr}\left[\ln\left(1 + e^{-\tilde{H}}\right)\right]. \end{aligned} \tag{S10}$$

Finally, we rewrite Eq. (S10) in terms of m defined in Eq. (S9)

$$\tilde{H} = \ln(1 - m) - \ln(m), \tag{S11}$$

to arrive at the expression

$$\begin{aligned} S_{\text{ent}} &= -\text{Tr}(\rho_{\text{sub}} \ln \rho_{\text{sub}}) = \text{Tr}\left[m \ln(1 - m) - m \ln(m)\right] - \text{Tr}\left[\ln(1 - m)\right] \\ &= -\text{Tr}\left[(1 - m)\ln(1 - m) + m \ln(m)\right] \end{aligned} \tag{S12}$$

that bridges the EE with the matrix m. Notice that Eq. (S12) is the general expression for the EE in non-interacting subsystems with the density matrix Eq. (S7) and the corresponding m matrix defined in Eq. (S9).

In our case, after turning on three QPCs, the m matrix becomes M for the subsystem A (discussed in S1A). We then arrive at the following expression for the EE:

$$S_{\text{ent}} = -\text{Tr}\left[(1 - M)\ln(1 - M) + M \ln M\right]. \tag{S13}$$

Equations (S5) and (S13) build the connection between FCS and EE in an HOM interferometer.

S1C. Calculating SEE from the generating function of FCS

A combination of Eqs. (S5) and (S13) successfully bridges the system EE and the generating function of FCS. These two equations are extensions of that of Refs. [S1, S2]: now the EE and the related FCS generating function are defined for subsystems A and B, rather than for two single channels. After scattering at two diluters, distribution functions become double-step-like, as shown in Fig. 1C of the main text, where the heights of middle steps respectively equal the transmission probabilities $\mathcal{T}_A(\epsilon)$ and $\mathcal{T}_B(\epsilon)$ at two diluters. Here we assume that both sources $\mathcal{S}_A$ and $\mathcal{S}_B$ are biased at the same voltage V_{bias}. Actually, when two biases are unequal, contributions from particles with energies between two biases will be removed following the definition of the entanglement pointer and the SEE.

Since Eqs. (S5) and (S13) share the form with that of Refs. [S1–S3], the EE can be calculated via the integral form

$$S_{\text{ent}} = \frac{1}{\pi}\int_{-\infty}^{\infty} du \frac{u}{\cosh^2 u} \text{Im}\left[\ln \chi(\pi - \eta - 2iu)\right], \tag{S14}$$

where η is a positive infinitesimal introduced to avoid possible divergence. The generating function of FCS of our system can be calculated following Ref. [S4]

$$\ln \chi(\lambda_{\text{FCS}}) = \frac{\tau}{h}\int_0^{eV_{\text{bias}}} d\epsilon \ln\left\{1 + \mathcal{T}\left[\mathcal{T}_A(1-\mathcal{T}_B)\left(e^{i\lambda_{\text{FCS}}} - 1\right) + \mathcal{T}_B(1-\mathcal{T}_A)\left(e^{-i\lambda_{\text{FCS}}} - 1\right)\right]\right\}, \tag{S15}$$

where h is the Planck constant and τ refers to the measuring time. In Eq. (S15), we have used the fact that the non-equilibrium distributions $\delta f_A(\epsilon) = \mathcal{T}_A$ and $\delta f_B(\epsilon) = \mathcal{T}_B$ for energies $0 < \epsilon < eV_{\text{bias}}$, and equals zero otherwise.

With Eqs. (S14) and (S15), the EE of our system becomes

$$\int_{u_0}^{\infty} du \frac{u}{\cosh^2 u} = \ln\left[2\cosh(u_0)\right] - u_0 \tanh(u_0) \tag{S16}$$

where u_0 is the solution of

$$\alpha_{12}(u_0) \equiv 1 - \mathcal{T}\mathcal{T}_A(1-\mathcal{T}_B)(1 + e^{2u_0}e^{-i\delta}) - \mathcal{T}\mathcal{T}_B(1-n_A)(1+e^{-2u_0}e^{i\delta}) = 0. \tag{S17}$$

Equation above has two solutions

$$\begin{aligned} u_1(\mathcal{T}_A,\mathcal{T}_B) &= \frac{1}{2}\ln\left[\frac{1 - a_1 - a_2 + \sqrt{(a_1+a_2-1)^2 - 4a_1a_2}}{2a_1}\right] \\ u_2(\mathcal{T}_A,\mathcal{T}_B) &= \frac{1}{2}\ln\left[\frac{1 - a_1 - a_2 + \sqrt{(a_1+a_2-1)^2 - 4a_1a_2}}{2a_2}\right], \end{aligned} \tag{S18}$$

where $a_1 = \mathcal{T}\mathcal{T}_A(1-\mathcal{T}_B)$ and $a_2 = \mathcal{T}\mathcal{T}_B(1-\mathcal{T}_A)$. With this notation, the entire EE (no interaction) comprises statistical contributions and contributions from single-source beams. It is given by

$$S_{\text{ent}}(\mathcal{T}_A,\mathcal{T}_B) = \frac{\tau}{h}\int d\epsilon \sum_{i=1,2}\left\{\ln\left[2\cosh u_i(\mathcal{T}_A,\mathcal{T}_B)\right] - u_i(\mathcal{T}_A,\mathcal{T}_B)\tanh[u_i(\mathcal{T}_A,\mathcal{T}_B)]\right\}. \tag{S19}$$

Expanding Eq. (S19) to the leading order of the dilutions $\mathcal{T}_A$ and $\mathcal{T}_B$, the zero-temperature S_{ent} of a non-interacting system approximately equals

$$S_{\text{ent}}(\mathcal{T}_A,\mathcal{T}_B,\epsilon)h/(e\tau V_{\text{bias}}) \approx \mathcal{T}\left[\mathcal{T}_A+\mathcal{T}_B-\mathcal{T}_A\ln(\mathcal{T}_A\mathcal{T})-\mathcal{T}_B\ln(\mathcal{T}_B\mathcal{T})\right]-\frac{1}{2}\mathcal{T}^2(\mathcal{T}_A^2+\mathcal{T}_B^2) \\ +\mathcal{T}(1-\mathcal{T})\mathcal{T}_A\mathcal{T}_B\ln(\mathcal{T}^2\mathcal{T}_A\mathcal{T}_B)+O(\mathcal{T}_{A,B}^3). \tag{S20}$$

Notice that the terms of the first line contain only single-particle contributions. These terms are removed once we calculate the statistics-induced EE. Performing the integration over energy, we arrive at Eq. (10) in Methods.

Inserting Eq. (S19) into Eq. (4) of the main text, we obtain an expression for the SEE,

$$S_{\text{SEE}} = -\frac{\tau}{h}\int d\epsilon\,\{\ln[2\cosh u_1(\mathcal{T}_A,\mathcal{T}_B)]-u_1(\mathcal{T}_A,\mathcal{T}_B)\tanh u_1(\mathcal{T}_A,\mathcal{T}_B) \\ +\ln[2\cosh u_2(\mathcal{T}_A,\mathcal{T}_B)]-u_2(\mathcal{T}_A,\mathcal{T}_B)\tanh u_2(\mathcal{T}_A,\mathcal{T}_B) \\ +\mathcal{T}_A\mathcal{T}\ln(\mathcal{T}_A\mathcal{T})+(1-\mathcal{T}_A\mathcal{T})\ln(1-\mathcal{T}_A\mathcal{T})+\mathcal{T}_B\mathcal{T}\ln(\mathcal{T}_B\mathcal{T})+(1-\mathcal{T}_B\mathcal{T})\ln(1-\mathcal{T}_B\mathcal{T})\}\,. \tag{S21}$$

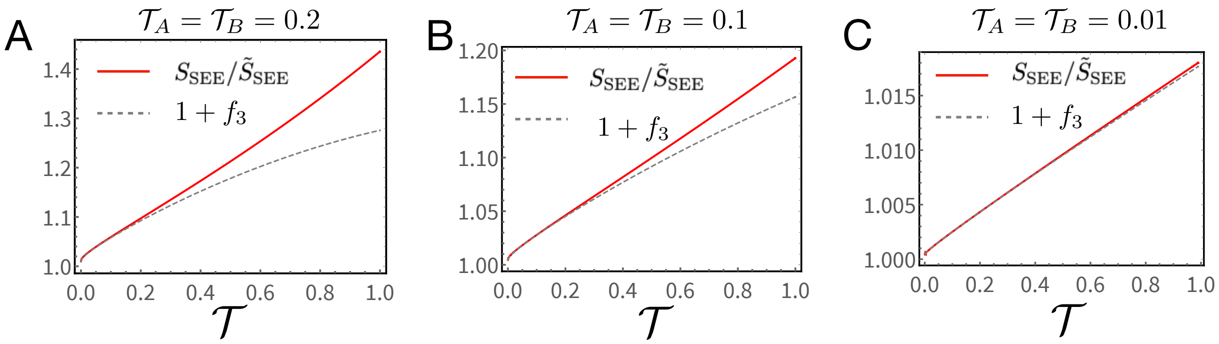

FIG. S1. The ratio between $\tilde{S}_{\text{SEE}}$ and S_{SEE} for different dilutions: A. $\mathcal{T}_A=\mathcal{T}_B=0.2$, B. $\mathcal{T}_A=\mathcal{T}_B=0.1$ and C. $\mathcal{T}_A=\mathcal{T}_B=0.01$. With a stronger dilution, the expansion to the third order (i.e., $1+f_3$) agrees better with the exact result of $\tilde{S}_{\text{SEE}}/S_{\text{SEE}}$.

We can further expand the SEE in powers of the diluters transmission coefficients, leading to

$$S_{\text{SEE}} \approx -\frac{eV_{\text{bias}}}{h}\mathcal{T}(1-\mathcal{T})\mathcal{T}_A\mathcal{T}_B\ln(\mathcal{T}^2\mathcal{T}_A\mathcal{T}_B)\left[1+f_3(\mathcal{T},\mathcal{T}_A,\mathcal{T}_B)\right], \tag{S22}$$

where the first part, $-eV_{\text{bias}}\mathcal{T}(1-\mathcal{T})\mathcal{T}_A\mathcal{T}_B\ln(\mathcal{T}^2\mathcal{T}_A\mathcal{T}_B)/h$, is the leading-order SEE [i.e., Eq. (11) of the main text] for strong dilution. This term is proportional to the product of the diluters transmission coefficients. The factor

$$f_3(\mathcal{T},\mathcal{T}_A,\mathcal{T}_B) \equiv \frac{\mathcal{T}_A+\mathcal{T}_B}{2}\frac{-1+3\mathcal{T}+2\mathcal{T}\ln(\mathcal{T}^2\mathcal{T}_A\mathcal{T}_B)}{\ln(\mathcal{T}^2\mathcal{T}_A\mathcal{T}_B)} \tag{S23}$$

refers to corrections from the higher-order terms in the diluters transmission coefficients. With this correction included, we plot the ratio of $\tilde{S}_{\text{SEE}}$ and S_{SEE} in Fig. S1 (red solid line). This ratio calculated for $\mathcal{T}=0.53$ and $\mathcal{T}_A=\mathcal{T}_B=0.21$ is the factor we use to rescale the SEE data in Fig. 4E of the main text. For any given values of $\mathcal{T}$, $\mathcal{T}_A$, $\mathcal{T}_B$, this ratio $\tilde{S}_{\text{SEE}}/S_{\text{SEE}}$ is well determined theoretically, and thus can always be used for the rescaling of SEE data. We also compare the exact ratio $\tilde{S}_{\text{SEE}}/S_{\text{SEE}}$ with the leading-order approximation $1+f_3(\mathcal{T},\mathcal{T}_A,\mathcal{T}_B)$ (the dashed line) defined in Eq. (S23). In agreement with our anticipation, the approximated value $1+f_3(\mathcal{T},\mathcal{T}_A,\mathcal{T}_B)$ agrees well with the ratio

$\tilde{S}_{\text{SEE}}/S_{\text{SEE}}$, when both $\mathcal{T}_A, \mathcal{T}_B$ are not too large (the difference between the solid and dashed curves is about 5% for the experimental values of the parameters); note that this approximation becomes even better with decreasing $\mathcal{T}$.

With interaction involved, the major structure of the result Eq. (S21) remains valid, after replacing $\mathcal{T}_A$ and $\mathcal{T}_B$ by the effective distribution functions $\tilde{f}_A$ and $\tilde{f}_B$ [see Eq. (S58) of Sec. S2 for details].

S1D. Rescaling the experimental data of SEE

In Fig. 5E of the main text, we compare the theoretical SEE with experimental data points. Since transmission coefficients of the diluters $\mathcal{T}_A = \mathcal{T}_B = 0.2$ are not small enough, $\tilde{S}_{\text{SEE}}$ obtained from the experimental data on the EP cannot precisely represent the real SEE, S_{SEE} (see Fig. 4B of the main text to see the difference between $\tilde{S}_{\text{SEE}}$ and S_{SEE}). To compare the experimental SEE with the theoretical curves, we thus rescale $\tilde{S}_{\text{SEE}}$ of the data, following the $S_{\text{SEE}} - \tilde{S}_{\text{SEE}}$ difference shown in Fig. 4B.

More specifically, experimental data is obtained with transmission amplitudes $\mathcal{T}_A = \mathcal{T}_B = 0.2$ and $\mathcal{T} = 0.53$. Following Fig. 4B of the main text, and Fig. S2, with these transmission parameters, $S_{\text{SEE}}/\tilde{S}_{\text{SEE}} \approx 1.22$. We thus rescale $\tilde{S}_{\text{SEE}}$ of the experimental data by the ratio above, to obtain the experimental values of S_{SEE} in Fig. 5E of the main text.

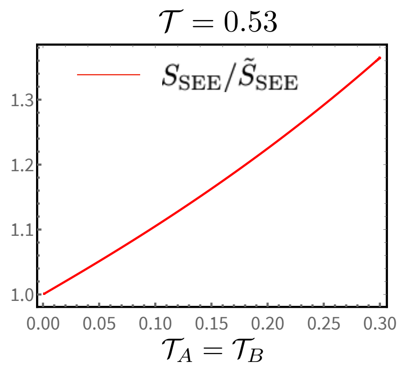

FIG. S2. The ratio of S_{SEE} and $\tilde{S}_{\text{SEE}}$ as a function of $\mathcal{T}_A = \mathcal{T}_B$, when $\mathcal{T} = 0.53$.

S2. INFLUENCE OF COULOMB INTERACTIONS

S2A. Influence of Coulomb interaction along the transport on the entanglement pointer

In real experiments, a sample with filling factor one might suffer from non-linearity that increases the complexity of measurement and theoretical analysis (cf. Sec. S5B). The samples with higher filling factors are more suitable in this respect. However, in such samples the inter-channel interactions are present, which should be taken into consideration. In this section, we consider a system with the filling factor 2, shown in Fig. S3, where each edge hosts two chiral channels. Only the outer channels (i.e., channels $A1$ and $B1$) receive signals that are diluted at two diluters located at $x = 0$, with the corresponding transmission probabilities $\mathcal{T}_A$ and $\mathcal{T}_B$, respectively. Only fermions in

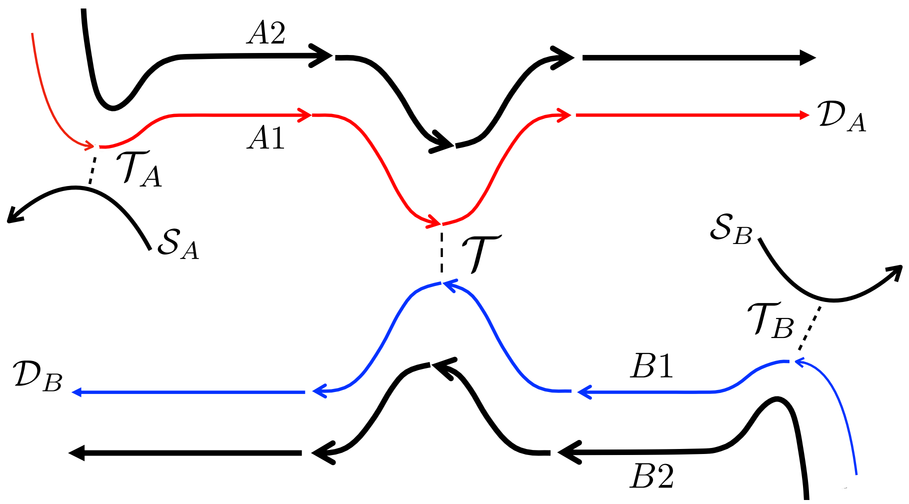

FIG. S3. The integer quantum Hall sample that hosts two chiral channels at each edge. Currents in outer channels is diluted at quantum point contacts (QPCs) with transmission probabilities $\mathcal{T}_A$ and $\mathcal{T}_B$. At the central QPC with the transmission $\mathcal{T}$, only the outer edges $A1$ and $B1$ communicate. The central QPC is located at the position $x = l$, with the distance l to two diluters. Only current of two outer channels are measured at drains $\mathcal{D}_A$ and $\mathcal{D}_B$.

channels $A1$ and $B1$ tunnel through the central QPC (located at $x = l$) with the transmission probability $\mathcal{T}$, through which a non-vanishing entanglement between two subsystems (the upper subsystem $A1$, $A2$, and the lower subsystem $B1$, $B2$) is established.

Fermions in the inner two channels (i.e., $A2$ and $B2$), in contrast, pass through two diluters with full transmission, and cannot directly witness the presence of the central QPC. They influence our results via the Coulomb interaction with fermions in the two outer channels. In this section, we consider only the interaction along the middle arms of the device. The influence of interaction in the central QPC will be included in Sec. S2C.

The above assumptions lead to the following system Hamiltonian

$$\begin{aligned} H_0 &= \sum_{\alpha=A,B,j=1,2} \frac{v_F}{4\pi} \int dx \left(\partial_x \phi_{\alpha j}\right)^2 , \quad H_{\text{int}} = \frac{v}{2\pi} \sum_{\alpha=A,B} \int dx \left(\partial_x \phi_{\alpha 1}\right)\left(\partial_x \phi_{\alpha 2}\right), \\ H_{\text{T}} &= t\left[\psi^\dagger_{A1}(l)\psi_{B1}(l) + \psi^\dagger_{B1}(l)\psi_{A1}(l)\right], \\ H_{\text{source}} &= t_A\left[\psi^\dagger_{A1}(0)\psi_{sA}(0) + \psi^\dagger_{sA}(0)\psi_{A1}(0)\right] + t_B\left[\psi^\dagger_{B1}(0)\psi_{sB}(0) + \psi^\dagger_{sB}(0)\psi_{B1}(0)\right], \end{aligned} \tag{S24}$$

where v_F is the Fermi velocity of free fermions, v is the interaction amplitude, and the phase ϕ_α is introduced through bosonization

$$\psi_\alpha = \frac{F_\alpha}{\sqrt{2\pi a}} e^{i\phi_\alpha} \tag{S25}$$

of the fermion in channel α. Here F_α is the Klein factor, and a is the short-distance cutoff. Operators ψ_{sA} and ψ_{sB} refer to fermions in sources $\mathcal{S}_A$ and $\mathcal{S}_B$, respectively.

Following Ref. [S5], with inter-channel interactions present, $H_0 + H_{\text{int}}$ becomes diagonal

$$H = \frac{v_+}{4\pi} \int dx \left[\left(\partial_x \phi_{\mathbf{A}+}\right)^2 + \left(\partial_x \phi_{\mathbf{S}+}\right)^2\right] + \frac{v_-}{4\pi} \int dx \left[\left(\partial_x \phi_{\mathbf{A}-}\right)^2 + \left(\partial_x \phi_{\mathbf{S}-}\right)^2\right], \tag{S26}$$

in terms of fast $(+)$ and slow $(-)$ fields

$$\begin{aligned} \phi_{\mathbf{A}\pm} &= \pm\frac{1}{2}\left[(\phi_{A1}-\phi_{B1})\pm(\phi_{A2}-\phi_{B2})\right], \\ \phi_{\mathbf{S}\pm} &= \pm\frac{1}{2}\left[(\phi_{A1}+\phi_{B1})\pm(\phi_{A2}+\phi_{B2})\right], \end{aligned} \tag{S27}$$

that travel with different velocities $v_\pm = v_F \pm v$. As a consequence, when an electron enters e.g., channel $A1$, it fractionalizes into two modes: a charge mode that travels at v_+, and a neutral (or dipole) mode that travels at v_-. By fractionalization, we refer to the spatial separation of these two modes after their propagation along the arms (see Methods and the Animation). In the S and A modes, fields in the upper ($A1$ or $A2$) two arms have the same and different signs, respectively, as those of the corresponding lower ($B1$ or $B2$) ones. With fields defined in Eq. (S27), the relation $\phi_{A1}-\phi_{B1} = \phi_{\mathbf{A}+}-\phi_{\mathbf{A}-}$ holds, and the tunneling operator transforms as

$$\psi^\dagger_{B1}\psi_{A1} \to \psi^\dagger_{\mathbf{A}-}\psi_{\mathbf{A}+}. \tag{S28}$$

Here,

$$\psi_{\mathbf{A}\pm} \sim \exp(i\phi_{\mathbf{A}\pm})/\sqrt{2\pi a}$$

refers to the operator after refermionization. Importantly, as $\psi_{\mathbf{A}\pm}$ are effectively non-interacting, their scattering at the central QPC can be studied with the scattering matrix formalism, with the scattering matrix

$$\begin{pmatrix} \psi_{\mathbf{A}+}(l^+) \\ \psi_{\mathbf{A}-}(l^+) \end{pmatrix} = \begin{pmatrix} r & -it \\ -it & r \end{pmatrix} \begin{pmatrix} \psi_{\mathbf{A}+}(l^-) \\ \psi_{\mathbf{A}-}(l^-) \end{pmatrix} \tag{S29}$$

that relates the fields right before ($l^- \equiv l-\eta$) and after ($l^+ \equiv l+\eta$) the scattering at the central QPC located at $x = l$ (η is again a positive infinitesimal). In Eq. (S29), we take $t = \sqrt{\mathcal{T}}$ and $r = \sqrt{1-\mathcal{T}} = \sqrt{\mathcal{R}}$ as real numbers. In this scattering matrix, t refers to the tunneling amplitude between states labeled $\mathbf{A}_+$ and $\mathbf{A}_-$. Following discussions in Ref. [S5], it also equals the tunneling amplitude between channels $A1$ and $B1$.

To calculate the current correlation function, we write down the current operators

$$\begin{aligned} I_{A1}(l^+) &= i[H, Q_{A1}(x>l^+)] = \frac{e}{2\pi}\left[v_F\partial_x\phi_{A1}(l^+) + v\partial_x\phi_{A2}(l^+)\right], \\ I_{B1}(l^+) &= i[H, Q_{B1}(x>l^+)] = \frac{e}{2\pi}\left[v_F\partial_x\phi_{B1}(l^+) + v\partial_x\phi_{B2}(l^+)\right], \end{aligned} \tag{S30}$$

after the central QPC, in terms of the fields before the rotation. Here $Q_{A1}(l^+)$ and $Q_{B1}(l^+)$ are the operators for the particle numbers corresponding to charges after the central QPC, in channels $A1$ and $B1$, respectively. Similar treatment of interaction and equation-of-motion method were used in Refs. [S6–S8].

To proceed, we write Eq. (S30) in terms of the non-interacting fields (S27), through which one can easily relate the operators before and after the central QPC with the scattering matrix Eq. (S29). After that, we transform back to

the original basis, through which current operators after the central QPC become

$$
\begin{aligned}
I_{A1}(l^+) &= \frac{e}{2\pi}\left\{v_F\mathcal{R}\partial_x\phi_{A1}(l^-)+ v_F\mathcal{T}\partial_x\phi_{B1}(l^-)+v\partial_x\phi_{A2}(l^-)\right.\\
&\left.+i\frac{v_F}{a}\sqrt{\mathcal{T}\mathcal{R}}\exp[-i\phi_{A1}(l^-)+i\phi_{B1}(l^-)]-i\frac{v_F}{a}\sqrt{\mathcal{T}\mathcal{R}}\exp[i\phi_{A1}(l^-)-i\phi_{B1}(l^-)]\right\},\\
I_{B1}(l^+) &= \frac{e}{2\pi}\left\{v_F\mathcal{T}\partial_x\phi_{A1}(l^-)+ v_F\mathcal{R}\partial_x\phi_{B1}(l^-)+v\partial_x\phi_{B2}(l^-)\right.\\
&\left.-i\frac{v_F}{a}\sqrt{\mathcal{T}\mathcal{R}}\exp[-i\phi_{A1}(l^-)+i\phi_{B1}(l^-)]+i\frac{v_F}{a}\sqrt{\mathcal{T}\mathcal{R}}\exp[i\phi_{A1}(l^-)-i\phi_{B1}(l^-)]\right\},
\end{aligned}
\tag{S31}
$$

in terms of bosonic operators before the central QPC. The first lines of both I_{A1} and I_{B1} consist of density operators that can be expressed in terms of current operators in front of the central QPC. The second lines in expressions (S31), on the other hand, display the quantum feature of transport at the central QPC: these operators produce finite contribution to the noise but zero contribution to the current averages. Using Eqs. (S31), we arrive at the time-dependent correlation function in the presence of interaction and obtain the difference of correlation functions entering the EP:

$$
\begin{aligned}
&\langle I_{A1}(l^+,t)I_{B1}(l^+,0)\rangle\Big|_{\mathcal{T}_A,\mathcal{T}_B}-\langle I_{A1}(l^+,t)I_{B1}(l^+,0)\rangle\Big|_{\mathcal{T}_A,0}-\langle I_{A1}(l^+,t)I_{B1}(l^+,0)\rangle\Big|_{0,\mathcal{T}_B}\\
&=\frac{e^2}{4\pi^2}\Bigg\{v_F^2\mathcal{T}\mathcal{R}\langle\partial_x\phi_{A1}(l^-,t)\partial_x\phi_{A1}(l^-,0)\rangle+v_F^2\mathcal{T}\mathcal{R}\langle\partial_x\phi_{B1}(l^-,t)\partial_x\phi_{B1}(l^-,0)\rangle\\
&+vv_F\mathcal{T}\langle\partial_x\phi_{A2}(l^-,t)\partial_x\phi_{A1}(l^-,0)\rangle+vv_F\mathcal{T}\langle\partial_x\phi_{B1}(l^-,t)\partial_x\phi_{B2}(l^-,0)\rangle\\
&-\frac{v_F^2}{a^2}\mathcal{T}\mathcal{R}\Big[\langle e^{-i\phi_{A1}(l^-,t)}e^{i\phi_{A1}(l^-,0)}\rangle\Big|_{\mathcal{T}_A,\mathcal{T}_B}\langle e^{i\phi_{B1}(l^-,t)}e^{-i\phi_{B1}(l^-,0)}\rangle\Big|_{\mathcal{T}_A,\mathcal{T}_B}\\
&+\langle e^{i\phi_{A1}(l^-,t)}e^{-i\phi_{A1}(l^-,0)}\rangle\Big|_{\mathcal{T}_A,\mathcal{T}_B}\langle e^{-i\phi_{B1}(l^-,t)}e^{i\phi_{B1}(l^-,0)}\rangle\Big|_{\mathcal{T}_A,\mathcal{T}_B}\Big]\Bigg\}\\
&-\frac{e^2}{4\pi^2}\Bigg\{v_F^2\mathcal{T}\mathcal{R}\langle\partial_x\phi_{A1}(l^-,t)\partial_x\phi_{A1}(l^-,0)\rangle+vv_F\mathcal{T}\langle\partial_x\phi_{A2}(l^-,t)\partial_x\phi_{A1}(l^-,0)\rangle\\
&-\frac{v_F^2}{a^2}\mathcal{T}\mathcal{R}\Big[\langle e^{-i\phi_{A1}(l^-,t)}e^{i\phi_{A1}(l^-,0)}\rangle\Big|_{\mathcal{T}_A,0}\langle e^{i\phi_{B1}(l^-,t)}e^{-i\phi_{B1}(l^-,0)}\rangle\Big|_{\mathcal{T}_A,0}\\
&+\langle e^{i\phi_{A1}(l^-,t)}e^{-i\phi_{A1}(l^-,0)}\rangle\Big|_{\mathcal{T}_A,0}\langle e^{-i\phi_{B1}(l^-,t)}e^{i\phi_{B1}(l^-,0)}\rangle\Big|_{\mathcal{T}_A,0}\Big]\Bigg\}\\
&-\frac{e^2}{4\pi^2}\Bigg\{v_F^2\mathcal{T}\mathcal{R}\langle\partial_x\phi_{B1}(l^-,t)\partial_x\phi_{B1}(l^-,0)\rangle+vv_F\mathcal{T}\langle\partial_x\phi_{B2}(l^-,t)\partial_x\phi_{B1}(l^-,0)\rangle\\
&-\frac{v_F^2}{a^2}\mathcal{T}\mathcal{R}\Big[\langle e^{-i\phi_{A1}(l^-,t)}e^{i\phi_{A1}(l^-,0)}\rangle\Big|_{0,\mathcal{T}_B}\langle e^{i\phi_{B1}(l^-,t)}e^{-i\phi_{B1}(l^-,0)}\rangle\Big|_{0,\mathcal{T}_B}\\
&+\langle e^{i\phi_{A1}(l^-,t)}e^{-i\phi_{A1}(l^-,0)}\rangle\Big|_{0,\mathcal{T}_B}\langle e^{-i\phi_{B1}(l^-,t)}e^{i\phi_{B1}(l^-,0)}\rangle\Big|_{0,\mathcal{T}_B}\Big]\Bigg\}.
\end{aligned}
\tag{S32}
$$

.

As a reminder, Eq. (S32) leads to our defined EP after the integral over time. The terms in red and blue of Eq. (S32) are correlations between operators from the same source: from S_A and S_B, respectively. They cancel out following

the definition of the EP [Eq. (1) of the main text]. The other terms become

$$
\begin{aligned}
-\frac{e^2}{h}\mathcal{T}(1-\mathcal{T})\int d\epsilon\ \{ & f_{A1}(l^-,\mathcal{T}_A,\epsilon)\left[1-f_{B1}(l^-,\mathcal{T}_B,\epsilon)\right]+f_{B1}(l^-,\mathcal{T}_B,\epsilon)\left[1-f_{A1}(l^-,\mathcal{T}_A,\epsilon)\right] \\
& -f_{A1}(l^-,\mathcal{T}_A,\epsilon)\left[1-f_{B1}(l^-,0,\epsilon)\right]-\left[1-f_{B1}(l^-,0,\epsilon)f_{A1}(l^-,\mathcal{T}_A,\epsilon)\right] \\
& -f_{A1}(l^-,0,\epsilon)\left[1-f_{B1}(l^-,\mathcal{T}_B,\epsilon)\right]-f_{B1}(l^-,\mathcal{T}_B,\epsilon)\left[1-f_{A1}(l^-,0,\epsilon)\right]\}
\end{aligned}
\tag{S33}
$$

after the integration over time, where

$$
f_\alpha(l^-,\mathcal{T}_\alpha,\epsilon)=v_F\int dte^{-i\epsilon t}\langle\psi^\dagger_\alpha(l^-,t)\psi_\alpha(l^-,0)\rangle\Big|_{\mathcal{T}_\alpha}
\tag{S34}
$$

refers to the distribution evaluated in channel α, with the corresponding transmission probability $\mathcal{T}_\alpha$.

A reorganization of (S33) gives us the EP of the interacting situation

$$
\mathcal{P}_\mathrm{E}=2\frac{e^2}{h}\mathcal{T}(1-\mathcal{T})\int d\epsilon\,\delta f_{A1}(l^-,\mathcal{T}_A,\epsilon)\delta f_{B1}(l^-,\mathcal{T}_B,\epsilon),
\tag{S35}
$$

which depends on the non-equilibrium parts of particle distributions,

$$
\delta f_{A1}(l^-,\mathcal{T}_A,\epsilon)=f_{A1}(l^-,\mathcal{T}_A,\epsilon)-f_{A1}(l^-,0,\epsilon),\qquad \delta f_{B1}(l^-,\mathcal{T}_B,\epsilon)=f_{B1}(l^-,\mathcal{T}_B,\epsilon)-f_{B1}(l^-,0,\epsilon)
$$

in front of the central QPC. As has been stated in the main text, Eq. (S35) shares the basic structure of the version without interaction along the arms, i.e., Eq. (3) of the main text, after replacing the double-step distribution functions with δf_α.

Following this fact, we see two important pieces of information on the effect (on the EP) of interaction along the arms. As the first message, the influence of interaction on the EP becomes negligible once the distance between the fast and slow wave packets from the same fermion is much smaller than the distance between the diluters and the central QPC: in this situation, the central QPC cannot feel the fractionalization of fermionic packets. Luckily, this is indeed the situation of our setup, with which we measure EP and the SEE [see Supplementary sections S2B and S2C, e.g., Fig. S4 and discussions around Eq. (S51) for details]. More importantly, Eq. (S35) tells us that even for the interacting situation, EP is a quantity that is determined by the two-particle scattering rate [proportional to $\int d\epsilon\delta f_{A1}(\epsilon)\delta f_{B1}(\epsilon)$], through which the statistical information is manifested.

S2B. Correlation functions with interaction present

In this section, we investigate the influence of interaction on correlation functions. We focus on the weak-interaction limit $|v|\ll v_F$. Following Eq. (S31), when expressing current operators after the central QPC in terms of that before the QPC, the operators consist of two categories of contributions, i.e., the current operators [e.g., $v_F\partial_x\phi_{A1}(l^-)$], and the vertex operators [e.g., $\psi^\dagger_{A1}(l^-)\psi_{B1}(l^-)$]. For later convenience, we define current operators

of these two categories, following

$$\begin{aligned}
I_{A1}^{\text{class}}(l^+) &= \frac{e}{2\pi}\left[v_F\mathcal{R}\partial_x\phi_{A1}(l^-)+ v_F\mathcal{T}\partial_x\phi_{B1}(l^-)+v\partial_x\phi_{A2}(l^-)\right],\\
I_{A1}^{\text{quant}}(l^+) &= i\frac{ev_F}{2\pi a}\sqrt{\mathcal{TR}}\left\{\exp[-i\phi_{A1}(l^-)+i\phi_{B1}(l^-)]-\exp[i\phi_{A1}(l^-)-i\phi_{B1}(l^-)]\right\},\\
I_{B1}^{\text{class}}(l^+) &= \frac{e}{2\pi}\left[v_F\mathcal{T}\partial_x\phi_{A1}(l^-)+ v_F\mathcal{R}\partial_x\phi_{B1}(l^-)+v\partial_x\phi_{B2}(l^-)\right],\\
I_{B1}^{\text{quant}}(l^+) &= -i\frac{ev_F}{2\pi a}\sqrt{\mathcal{TR}}\left\{\exp[-i\phi_{A1}(l^-)+i\phi_{B1}(l^-)]-\exp[i\phi_{A1}(l^-)-i\phi_{B1}(l^-)]\right\},
\end{aligned} \tag{S36}$$

through which the previous current operators can be written as $I_{A1}=I_{A1}^{\text{class}}+I_{A1}^{\text{quant}}$ and $I_{B1}=I_{B1}^{\text{class}}+I_{B1}^{\text{quant}}$. In Eq. (S36), we call current operators defined with density operators as classical and those with vertex operators as quantum, for two reasons. Firstly, the quantum terms do not contribute to the current averages after the QPC: indeed, they influence only the current correlation. More importantly, following Ref. [S9], the classical part reflects the noise produced at two diluters before the central QPC. This part of noise, after being produced at two diluters, simply propagates along the arms, before the current has arrived at the central QPC. The quantum part instead encodes the noise generated by the scattering at the central QPC. As another advantage of Eq. (S36), one quantum and one classical operators have a vanishing correlation. One can thus calculate correlation functions, e.g., $\langle I_{A1}I_{B1}\rangle$, by addressing the classical and quantum contributions individually.

To calculate the correlation of classical current operators, we further express the density operators in terms of the current operators before the QPC, leading to

$$\begin{aligned}
\partial_x\phi_{A1}(l^-) &= \frac{2\pi}{e}\frac{v_F I_{A1}(l^-)-vI_{A2}(l^-)}{v_F^2-v^2}, \quad \partial_x\phi_{A2}(l^-) = -\frac{2\pi}{e}\frac{vI_{A1}(l^-)-v_F I_{A2}(l^-)}{v_F^2-v^2},\\
\partial_x\phi_{B1}(l^-) &= \frac{2\pi}{e}\frac{v_F I_{B1}(l^-)-vI_{B2}(l^-)}{v_F^2-v^2}, \quad \partial_x\phi_{B2}(l^-) = -\frac{2\pi}{e}\frac{vI_{B1}(l^-)-v_F I_{B2}(l^-)}{v_F^2-v^2}.
\end{aligned} \tag{S37}$$

With expressions in Eq. (S37), the classical part of the current becomes

$$\begin{aligned}
I_{B1}^{\text{class}}(l^+) \equiv& \frac{e}{2\pi}\left\{v_F\mathcal{R}\partial_x\phi_{A1}(l^-)+ v_F\mathcal{T}\partial_x\phi_{B1}(l^-)+v\partial_x\phi_{A2}(l^-)\right\}\\
=&\frac{1}{v_+v_-}\left[(v_F^2\mathcal{R}-v^2)I_{A1}(l^-)+v_Fv\mathcal{T}I_{A2}(l^-)+v_F^2\mathcal{T}I_{B1}(l^-)-v_Fv\mathcal{T}I_{B2}(l^-)\right],\\
I_{B1}^{\text{class}}(l^+) \equiv& \frac{e}{2\pi}\left\{v_F\mathcal{T}\partial_x\phi_{A1}(l^-)+ v_F\mathcal{R}\partial_x\phi_{B1}(l^-)+v\partial_x\phi_{B2}(l^-)\right\}\\
=&\frac{1}{v_+v_-}\left[(v_F^2\mathcal{R}-v^2)I_{B1}(l^-)+v_Fv\mathcal{T}I_{B2}(l^-)+v_F^2\mathcal{T}I_{A1}(l^-)-v_Fv\mathcal{T}I_{A2}(l^-)\right].
\end{aligned} \tag{S38}$$

As a consequence of charge fractionalization, post-QPC current operators in $A1$ and $B1$ become dependent on current operators in all four arms, although direct tunneling from $A2$ and $B2$ to $A1$ and $B1$ are not allowed. However, in our system, currents from the sources only enter the middle two arms $A1$ and $B1$, leading to $\langle I_{A2}\rangle=\langle I_{B2}\rangle=0$. As a result, most contributions of $\langle I_{A1}^{\text{class}}(l^+,t)I_{B1}^{\text{class}}(l^+,0)\rangle_{\text{irr}}$ vanish, except for that from auto-correlations $\langle I_{A1}(l^-,t)I_{A1}(l^-,0)\rangle_{\text{irr}}$ and $\langle I_{B1}(l^-,t)I_{B1}(l^-,0)\rangle_{\text{irr}}$. Indeed, now the correlation function between the classical op-

erators becomes

$$\begin{aligned}\langle I^{\rm class}_{A1}(l^+,t) I^{\rm class}_{B1}(l^+,t)\rangle_{\rm irr} &= \frac{(v_F^2\mathcal{R}-v^2)v_F^2\mathcal{T}}{(v_F^2-v^2)^2}\left[\langle I_{A1}(l^-,t)I_{A1}(l^-,0)\rangle_{\rm irr}+\langle I_{B1}(l^-,t)I_{B1}(l^-,0)\rangle_{\rm irr}\right]\\ &\approx \mathcal{T}\mathcal{R}\left[1+\frac{v^2}{v_F^2}\left(2-\frac{1}{\mathcal{R}}\right)\right]\left[\langle I_{A1}(l^-,t)I_{A1}(l^-,0)\rangle_{\rm irr}+\langle I_{B1}(l^-,t)I_{B1}(l^-,0)\rangle_{\rm irr}\right],\end{aligned} \tag{S39}$$

where in the second line we have expanded the correlation to the leading order of interaction v^2/v_F^2. After the integration over time, the zero-frequency noise becomes

$$\begin{aligned}\int dt \langle I_{A1}(l^-,t)I_{A1}(l^-,0)\rangle_{\rm irr} &= \frac{e^3 V_{\rm bias}}{h}\mathcal{T}_A(1-\mathcal{T}_A),\\ \int dt \langle I_{B1}(l^-,t)I_{B1}(l^-,0)\rangle_{\rm irr} &= \frac{e^3 V_{\rm bias}}{h}\mathcal{T}_B(1-\mathcal{T}_B).\end{aligned} \tag{S40}$$

which does not depend on the amplitude of interaction v. With this knowledge, Eq. (S39) indicates that intra-edge interaction between neighboring channels generates a leading correction

$$\frac{e^3 V_{\rm bias}}{h}\mathcal{T}(1-\mathcal{T})\frac{v^2}{v_F^2}\left(2-\frac{1}{1-\mathcal{T}}\right)\left[\mathcal{T}_A(1-\mathcal{T}_A)+\mathcal{T}_B(1-\mathcal{T}_B)\right], \tag{S41}$$

to the current correlation $\langle I^{\rm class}_{A1}(l^+,t) I^{\rm class}_{B1}(l^+,0)\rangle_{\rm irr}$. The correction given by Eq. (S41) is positive if the transmission at the central QPC $\mathcal{T} < 1/2$, and becomes negative if $\mathcal{T} > 1/2$. Notice that this interaction-induced correction to the cross-correlation function is linear in the dilution $\mathcal{T}_A$ or $\mathcal{T}_B$, and remains finite even with only one source on.

To understand how charge fractionalization introduces this extra correlation function, we remind ourselves that, following the effective free Hamiltonian (S26), two plus modes $\mathbf{A}_+$ and $\mathbf{S}_+$ are traveling with the speed v_+, which is larger than that (v_-) of two minus modes $\mathbf{A}_-$ and $\mathbf{S}_-$. In this basis, the currents carried by symmetric modes $\mathbf{S}_+$ and $\mathbf{S}_-$ are not affected by the central QPC [see Eq. (S28)]. By contrast, the wave packets of the other two modes $\mathbf{A}_+$ and $\mathbf{A}_-$ tunnel to each other at the central QPC. Most interestingly, when an $\mathbf{A}_\pm$ wave packet transmits into an $\mathbf{A}_\mp$ packet after scattering at the central QPC, the speed of the wave packet suddenly changes from $v_\pm$ to $v_\mp$. After including contributions of both asymmetric modes, the current transmitted (between $A1$ and $B1$) at the central QPC becomes larger than that in the non-interacting case. Indeed, with only the upper source on ($\mathcal{T}_B = 0$), classical current operators become

$$\begin{aligned}I^{\rm class}_{A1}(l^+) &\sim (1-\mathcal{T}_A-\mathcal{T}_v)I_{A1}(l^-),\\ I^{\rm class}_{B1}(l^+) &\sim (\mathcal{T}_A+\mathcal{T}_v)I_{A1}(l^-),\end{aligned} \tag{S42}$$

where we have neglected I_{A2} that does not contribute to the correlation function. In Eq. (S42), the dressed transmission

$$\mathcal{T}_v \equiv v^2\mathcal{T}/(v_F^2-v^2) > 0$$

is positive, indicating an extra amount of transmitted current. With these operators, the correlation becomes

$$\langle I^{\rm class}_{A1}(l^+) I^{\rm class}_{B1}(l^+)\rangle = \left[\mathcal{T}\mathcal{R}-(\mathcal{T}-\mathcal{R})\mathcal{T}_v-\mathcal{T}_v^2\right]\langle I_{A1}(l^-)I_{B1}(l^-)\rangle. \tag{S43}$$

The sign of the leading order correction ($\propto \mathcal{T}_v$) depends on the transmission amplitude $\mathcal{T}$, in agreement with Eq. (S41).

Although an extra amount of transmitted noise induces an extra classical noise, one cannot incorporate the interaction effect by a simple modification of the transmission $\mathcal{T}$. Indeed, as will be shown shortly, modifications on the correlation of the quantum part cannot be described by a modified $\mathcal{T}$.

Now we evaluate the correlation of the quantum part, i.e., vertex operators, by inspecting the correlation function $\langle\psi^\dagger_{A1}(l,t)\psi_{A1}(l,0)\rangle$ to the leading order of interaction v. Following Ref. [S10], one can calculate the correlation function by writing the fields

$$\begin{aligned}\phi_{A1}(l,t) &= -\frac{\pi}{e}[Q_{A1}(t-l/v_+)+Q_{A2}(t-l/v_+)+Q_{A1}(t-l/v_-)-Q_{A2}(t-l/v_-)],\\ \phi_{A2}(l,t) &= -\frac{\pi}{e}[Q_{A1}(t-l/v_+)+Q_{A2}(t-l/v_+)-Q_{A1}(t-l/v_-)+Q_{A2}(t-l/v_-)],\end{aligned}\tag{S44}$$

in terms of the operators $Q_{A1}(t)$ and $Q_{A2}(t)$,

$$Q_\alpha(t) = \int_{-\infty}^{t} dt' j_\alpha(t'),\tag{S45}$$

which refer to the number of charges that have entered the interfering arms at time t. Here, $j_\alpha(t)$ is the current operator at time t right after the diluter in arm α. In terms of the charge operators, the correlation of bosonized operators becomes

$$\begin{aligned}\langle e^{-i\phi_{A1}(t)}e^{i\phi_{A1}(0)}\rangle =&\left\langle e^{i\frac{\pi}{e}[Q_{A1}(t-l/v_+)+Q_{A1}(t-l/v_-)]}e^{-i\frac{\pi}{e}[Q_{A1}(-l/v_+)+Q_{A1}(-l/v_-)]}\right\rangle\\ \approx&\langle e^{2i\frac{\pi}{e}Q_{A1}(t-l/v_+)}e^{-2i\frac{\pi}{e}Q_{A1}(-l/v_+)}\rangle\left[1+\frac{4\pi^2l^2v^2}{e^2v_F^4}\langle j_{A1}(t)j_{A1}(0)\rangle_{\text{irr}}\right]\\ \approx&\langle e^{2i\frac{\pi}{e}Q_{A1}(t-l/v_+)}e^{-2i\frac{\pi}{e}Q_{A1}(-l/v_+)}\rangle\left(1-\frac{l^2v^2}{v_F^4t^2}\right).\end{aligned}\tag{S46}$$

From this expression, we see that interaction introduces a (negative) correction, i.e.,

$$\langle\psi^\dagger_{A1}(t)\psi_{A1}(0)\rangle \to \langle\psi^\dagger_{A1}(t)\psi_{A1}(0)\rangle\left(1-\frac{l^2v^2}{v_F^4t^2}\right),\tag{S47}$$

to the time-dependent correlation of vertex operators. This perturbative expansion is valid when $vl/v_F \ll v_F t \sim \lambda$, where $\lambda \sim ev_F/I$ describes the wave-packet width that depends on the amplitude of nonequilibrium current I. The factor vl/v_F is indeed the distance between the centers of two wave packets (charge and neutral modes) fractionalized from a single fermion.

For a more intuitive understanding of the validity of the perturbative expansion, we look at Fig. S4 for an interacting quantum Hall edge with filling factor 2. Here three length scales have been highlighted. Under the influence of interaction, a fermion that enters $A1$ fractionalizes into a charge and a dipole packets that travel at different velocities $v_\pm$, as illustrated by the two wave-packets (of width λ) in Fig. S4. When the faster mode (charge mode) has arrived at the QPC, the centers of these two packets are separated by the distance $d_{\text{pack}} \approx vl/v_F$. The importance of interaction is then determined by the relation between the distance between the packets d_{pack} and their width λ. Specifically, when $\lambda \gg d_{\text{pack}}$, the fermion can not sense its fractionalization, so that the interaction influence is negligible. On

the contrary, two modes are well-separated in the opposite limit $\lambda \ll d_{\text{pack}}$, and the effect of interaction becomes significant. Actually, in Eq. (S47), the factor that quantifies the significance of interaction, i.e., $vl/(v_F\lambda) = d_{\text{pack}}/\lambda$ becomes important only when $d_{\text{pack}} \gg \lambda$, in agreement with our qualitative analysis.

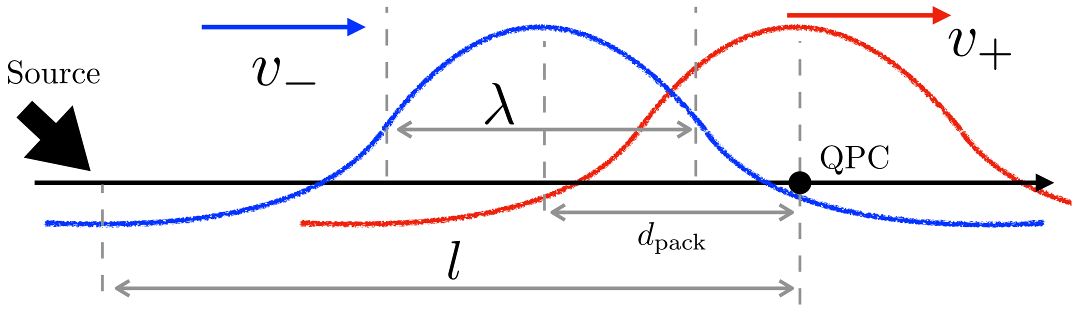

FIG. S4. Length scales of an interacting system. If $d_{\text{pack}} \ll \lambda$, two wave packets (red and blue) splitted from the same fermion becomes indistinguishable. Of this case, the interaction influences on entanglement pointer and statistics-induced entanglement entropy are weak.

To display the effect of interaction, we provide an experiment-theory comparison of cross-correlation functions in Figs. 5C and D of the main text, for the diluter transmission coefficients equal $\mathcal{T}_A = \mathcal{T}_B = 0.2$. Interaction generates extra cross-correlation that equals to

$$
\begin{aligned}
&\int dt \langle I_A(t) I_B(0)\rangle_{\text{irr}}\Big|_v - \int dt \langle I_A(t) I_B(0)\rangle_{\text{irr}}\Big|_{v=0} \\
&= \frac{e^2}{h}\mathcal{T}(1-\mathcal{T})\int d\epsilon \left(2 - \frac{1}{1-\mathcal{T}}\right)\frac{v^2}{v_F^2} \\
&\times \Big\{\delta f_{A1}(l^-,\mathcal{T}_A,\epsilon)[1-2f_{A1}(l^-,0,\epsilon) - \delta f_{A1}(l^-,\mathcal{T}_A,\epsilon)] + \delta f_{B1}(l^-,\mathcal{T}_B,\epsilon)[1-2f_{B1}(l^-,0,\epsilon) - \delta f_{B1}(l^-,\mathcal{T}_B,\epsilon)]\Big\} \\
&+ \frac{e^2}{h}\mathcal{T}(1-\mathcal{T})\int d\epsilon \,\frac{v^2}{v_F^2}\frac{l^2}{\lambda^2} \\
&\times \Big\{\delta f_{A1}(l^-,\mathcal{T}_A,\epsilon)[1-2f_{A1}(l^-,0,\epsilon)] + \delta f_{B1}(l^-,\mathcal{T}_B,\epsilon)[1-2f_{B1}(l^-,0,\epsilon)] - 2\delta f_{A1}(l^-,\mathcal{T}_A,\epsilon)\delta f_{B1}(l^-,\mathcal{T}_B,\epsilon)\Big\}.
\end{aligned}
\tag{S48}
$$

These modifications are incorporated in Sec. S2C, through the introduction of parameters P_A, P_B, and P_{frac}. Importantly, the linear-in-δf terms on the right-hand side of Eq. (S48) perfectly cancel when calculating the EP. The last term, which is quadratic in δf, induces a finite correction to the EP. It is, however, comparatively smaller than the linear-in-δf terms in the strongly diluted limit, as (i) it is proportional to the product of transmission probabilities at both diluters, i.e., $\mathcal{T}_A\mathcal{T}_B$, and (ii) it has a smaller prefactor $d_{\text{pack}}^2/\lambda^2$. The SEE also receives a minor influence from interactions, as it is proportional to the EP in the strongly diluted limit.

In cross-correlation functions, the linear terms $\mathcal{T}_A P_A$ and $\mathcal{T}_B P_B$ remain, and the interaction-induced corrections are rather manifest, as can be seen from Figs. 5C and D of the main text. Indeed, in the presence of interaction, correlation functions display apparent deviations from the non-interacting ones: the former ones fit significantly better (than the non-interacting ones) the experimental data. We have taken $v/v_F = 0.4$ and $l/\lambda = 0.5$ in this fitting.

The interaction-induced modification of the correlation functions is in strong contrast to the minor changes on the EP displayed in Fig. 5E of the main text. This comparatively much weaker sensitivity of the entanglement pointer to interactions originates from the cancellation of leading terms in Eq. (S48).

For later convenience, we can also write down the correction to the auto-correlation as

$$\begin{aligned}
&\int dt\langle I_A(t)I_A(0)\rangle_{\text{irr}}\Big|_v - \int dt\langle I_A(t)I_A(0)\rangle_{\text{irr}}\Big|_{v=0} \\
&= \frac{e^2}{h}\int d\epsilon\left(2-\frac{1}{1-\mathcal{T}}\right)\frac{v^2}{v_F^2} \\
&\times\Big\{\mathcal{T}^2\delta f_{A1}(l^-,\epsilon)[1-2f_{A1}(l^-,0,\epsilon)-\delta f_{A1}(l^-,\epsilon)]+(1-\mathcal{T})^2\delta f_{B1}(l^-,\epsilon)[1-2f_{B1}(l^-,0,\epsilon)-\delta f_{B1}(l^-,\epsilon)]\Big\} \\
&-\frac{e^2}{h}\mathcal{T}(1-\mathcal{T})\int d\epsilon\,\frac{v^2}{v_F^2}\frac{l^2}{\lambda^2} \\
&\times\Big\{\delta f_{A1}(l^-,\epsilon)[1-2f_{A1}(l^-,0,\epsilon)]+\delta f_{B1}(l^-,\epsilon)[1-2f_{B1}(l^-,0,\epsilon)]-2\delta f_{A1}(l^-,\epsilon)\delta f_{B1}(l^-,\epsilon)\Big\},
\end{aligned} \tag{S49}$$

which is of the same order as the interaction-induced correction to the cross-correlation. However, the correction Eq. (S49) is experimentally less visible in comparison to that of the cross-correlation, since the non-interacting auto-correlation is normally much larger than the interaction-induced correction Eq. (S49) in strongly diluted systems.

S2C. Parameterization of the interaction: Correlation functions, entanglement pointer and SEE with two sources of interaction

In experiments, all quantities, including correlation functions, EP, and SEE are influenced by two sources of interactions: the interaction along the arms and that at the central QPC. The former source of interaction, as has been shown in previous sections [more specifically in Eqs. (S48) and (S49)], can be obtained from the interaction-induced modifications of either the cross-correlation and autocorrelation functions. These modifications can be described by parameters P_A, P_B, and P_{frac} (introduced below) that have explicit connections to microscopic parameters under either a strong (see, e.g., Ref. [S9]) or weak (Sec. S2B) fractionalization. By contrast, the influence of interaction near the QPC is hard to evaluate from microscopic parameters, as the interaction depends on non-universal details of the sample, including e.g., the size and shape of the QPC, how strongly the QPC has been depleted, and also on whether the tunneling across the QPC is adiabatic or not (see e.g., Ref. [S11] as a recent example). Therefore, we investigate the influence of interaction at the central QPC by introducing a phenomenological parameter P_{QPC}: when P_{QPC} is positive (or negative), it indicates an extra bunching (or anti-bunching) probability of two-particle scatterings induced by the interaction at the central QPC. Fortunately, as will be shown in this section, interaction parameters of both types can be obtained from the theory-experiment comparison. Most importantly, we show that the leading interaction-induced effects on cross-correlations cancel out when calculating the EP and the corresponding SEE. Consequently, the EP and SEE are less sensitive to interactions and thus better (than the correlation functions) quantify the statistics-related entanglement of the system.

We begin by considering the effect of the phenomenologically introduced parameter $P_{\rm QPC}$. Briefly, as $P_{\rm QPC}$ is generated by two-particle scattering, it is only finite when both sources are on. For instance, for a fermionic system where particles only interact at the central QPC, the zero-temperature cross-correlation becomes

$$\int dt \langle I_A(t) I_B(0)\rangle_{\rm irr}\Big|_{v=0} = -\frac{e^3}{h}\mathcal{T}(1-\mathcal{T})\left[(\mathcal{T}_A-\mathcal{T}_B)^2+\mathcal{T}_A\mathcal{T}_B P_{\rm QPC}\right]V_{\rm bias}, \tag{S50}$$

where the second term is the correction from finite $P_{\rm QPC}$. The prefactor of $P_{\rm QPC}$ [i.e., $\mathcal{T}(1-\mathcal{T})\mathcal{T}_A\mathcal{T}_B$] is the probability that two distinguishable particles bunch after a two-particle scattering event. In Eq. (S50), only a positive $P_{\rm QPC}$, i.e., an extra interaction-induced bunching is allowed, as free fermions perfectly anti-bunch during two-particle scatterings. A positive value of $P_{\rm QPC}$ has indeed been shown numerically in, e.g., Ref. [S12], where fermions Coulomb-interact near the central QPC. This extra bunching can be understood as a spatial separation (of two incoming fermions) generated by the repulsive Coulomb interaction near the central QPC. By contrast, for bosons, interaction near the QPC would possibly induce an extra anti-bunching. It is then another non-trivial issue to build a confirmative connection between the interaction Hamiltonian and the value of $P_{\rm QPC}$ for different types of particles.

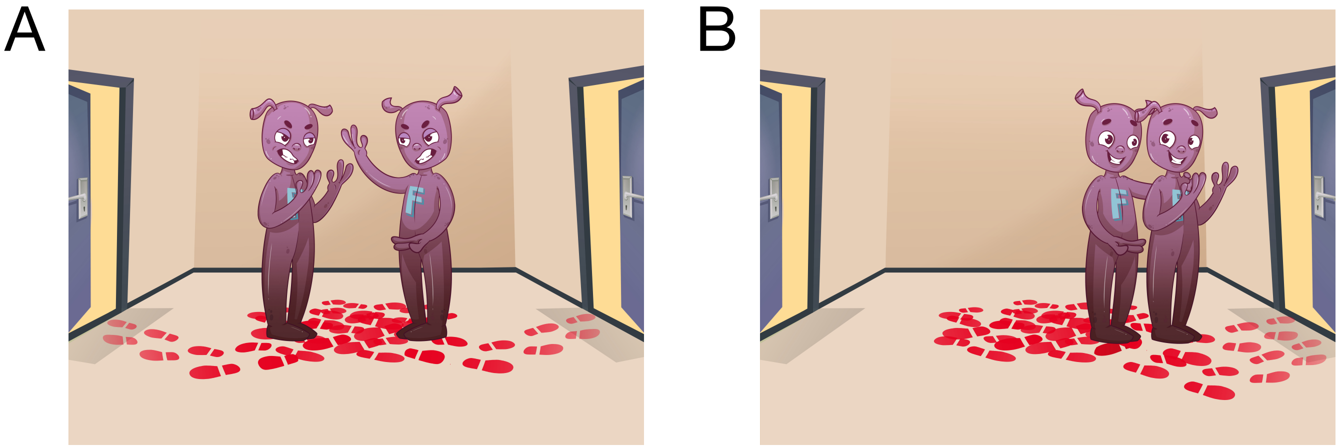

FIG. S5. Competing effects in entanglement: interaction-induced entanglement blurs statistics-induced entanglement. (A) When two aliens from the star "F" meet, they instinctively hate each other, and opt to leave the room through different doors (alluding to statistics-induced anti-bunching). (B) After communicating with each other (alluding to Coulomb interaction), the two "F"s may change their mutual sentiment, and decide to bunch together, leaving through the same door.

The following caricature (Fig. S5) illustrates two competing effects, particle statistics and interaction, in two-particle scatterings. Consider two alien civilizations, one from star "F" (where aliens hate each other) and the other from star "B" (where aliens like each other). Two aliens from the same star enter a meeting room from two opposite doors. Upon entering the room, they immediately recognize the other alien. The "F"s would like to leave through opposite doors (Fig. S5A), (the "B"s would naturally prefer leaving the room together through the same door). This would be a representation of instantaneously acquired statistical entanglement. However, if the two aliens spend some time in the meeting room and interact with each other (alluding to Coulomb interaction), their instinctive preference

(statistical entanglement) may be modified by interaction-acquired entanglement, affecting the probability of leaving bunched together [Fig. S5B, which is explicitly quantified by $P_{\rm QPC}$ of Eq. (S50)] (for the "B"s— anti-bunched). Similarly, the measured current-current correlations may manifest contributions (to the entanglement) from both statistics and Coulomb interactions. Following the experimental data, entanglement pointer of our model contains mainly contributions from statistics.

Although the value of $P_{\rm QPC}$ is generically non-universal and hard to evaluate theoretically, it can be extracted from the theory-experiment comparison, after including interactions both along the arms and at the central QPC. Similarly to $P_{\rm QPC}$ introduced for the interaction at the QPC, we can introduce extra factors to describe the influence of interactions along the arms. Indeed, following Eq. (S48), after including interactions along the arms, the zero-temperature cross-correlation function becomes (included in Methods)

$$\int dt\langle I_A(t)I_B(0)\rangle_{\rm irr} = -\frac{e^3}{h}\mathcal{T}(1-\mathcal{T})\left[(\mathcal{T}_A-\mathcal{T}_B)^2+\mathcal{T}_AP_A+\mathcal{T}_BP_B+\mathcal{T}_A\mathcal{T}_B(P_{\rm QPC}+P_{\rm frac})\right]V_{\rm bias}, \tag{S51}$$

where

$$\begin{aligned} P_A &= -\left[(1-\mathcal{T}_A)\left(1-\frac{1}{2-2\mathcal{T}}\right)+\frac{l^2}{\lambda^2}\right]\frac{v^2}{v_F^2},\\ P_B &= -\left[(1-\mathcal{T}_B)\left(1-\frac{1}{2-2\mathcal{T}}\right)+\frac{l^2}{\lambda^2}\right]\frac{v^2}{v_F^2}, \end{aligned} \tag{S52}$$

quantify the modification of the correlation function due to the particle fractionalization in each arm [the linear-in-δf terms in Eq. (S48)]. These two terms are finite as long as the corresponding source is on. By contrast, the other part $\mathcal{T}_A\mathcal{T}_BP_{\rm frac}$, with

$$P_{\rm frac} = 2\frac{v^2}{v_F^2}\frac{l^2}{\lambda^2}, \tag{S53}$$

is only finite when both sources are on [the last term in Eq. (S48)]. We further define

$$P_{\rm int} \equiv P_{\rm frac} + P_{\rm QPC} \tag{S54}$$

as the parameter that reflects double-source contributions to the correlation function. With these parameters and the definition [Eq. (1) of the main text], the EP of fermions now reads as

$$\mathcal{P}_{\rm E} = (2-P_{\rm int})\frac{e^3}{h}\mathcal{T}(1-\mathcal{T})\mathcal{T}_A\mathcal{T}_BV_{\rm bias}. \tag{S55}$$

In our case, as follows from the theory-experiment comparison, $P_A = P_B \approx -0.057$ and $P_{\rm int} \approx 0.08$ with the values of v/v_F and l/λ evaluated in Sec. S2 [see lines below Eq. (S48)]. In the strongly diluted limit, the single-source contributions $\mathcal{T}_AP_A$ and $\mathcal{T}_BP_B$ are generically much larger than the two-source contribution $\mathcal{T}_A\mathcal{T}_BP_{\rm int}$, as $\mathcal{T}_AP_A$ and $\mathcal{T}_BP_B$ are linear functions of the diluter transmission coefficients, while $\mathcal{T}_A\mathcal{T}_BP_{\rm int}$ is quadratic in diluters transmission. Meanwhile, the values of P_A and P_B are generically larger than $P_{\rm frac}$ from fractionalization, when $l \ll \lambda$ for strong enough dilution. Indeed, contributions from $\mathcal{T}_AP_A$ and $\mathcal{T}_BP_B$ are the leading terms that introduce the manifest modification of correlation functions in Figs. 5C and 5D of the main text. However, despite their leading importance for

correlation functions, $\mathcal{T}_A P_A$ and $\mathcal{T}_B P_B$ perfectly cancel out when calculating the entanglement pointer and the ensued SEE, as has been discussed after Eq. (S48). This cancellation, importantly, indicates that the entanglement pointer introduced in our work has much stronger resistance against interactions: the leading interaction-induced corrections to correlation functions cancel out in the EP, and a strong signal of statistics now becomes manifest.

The fact that P_{QPC} and P_{frac} only appear in two-source correlations helps the extraction of interaction parameters. Indeed, with the above knowledge, in experiments, we can first evaluate the values of P_A and P_B from single-source correlations, and equivalently obtain the value v along two interfering arms, together with the value of P_{frac}. Afterwards, one moves to the two-source correlation to obtain P_{QPC} that represents the effect of interaction at the central QPC. As shown in Figs. 5C and 5D of the main text, the interaction in the arm (i.e., v) suffices to produce remarkable theory-experiment matching, indicating a rather small value of $P_{\text{QPC}} \approx 0.02$ for our sample.

It is worth noticing that the interaction amplitudes v and P_{int} can also be obtained from the autocorrelation function. For instance, the autocorrelation of current operators in the upper arm, with interaction included, becomes [see Eq. (S49)]

$$
\begin{aligned}
\int dt \langle I_A(t) I_A(0)\rangle_{\text{irr}} = &\frac{e^3}{h} V_{\text{bias}} [\mathcal{T}_A(1-\mathcal{T}) + \mathcal{T}_B\mathcal{T}][1 - \mathcal{T}_A(1-\mathcal{T}) - \mathcal{T}_B\mathcal{T}] \\
&+ \frac{e^3}{h} V_{\text{bias}} \mathcal{T}(1-\mathcal{T})\mathcal{T}_A\mathcal{T}_B(P_{\text{QPC}} + P_{\text{frac}}) \\
&+ \frac{e^3}{h} V_{\text{bias}} \left[\mathcal{T}^2\mathcal{T}_A(1-\mathcal{T}_A) + (1-\mathcal{T})^2\mathcal{T}_B(1-\mathcal{T}_B)\right] \left(2 - \frac{1}{1-\mathcal{T}}\right) \frac{v^2}{v_F^2} \\
&- 2\frac{e^3}{h} V_{\text{bias}} \mathcal{T}(1-\mathcal{T})\left(\mathcal{T}_A + \mathcal{T}_B\right) \frac{v^2}{v_F^2}\frac{l^2}{\lambda^2}.
\end{aligned}
\tag{S56}
$$

Similarly to its role in cross-correlations, $P_{\text{int}} = P_{\text{QPC}} + P_{\text{frac}}$ shows up in Eq. (S56) of the autocorrelation only when both sources are on. One can then, in principle, evaluate the values of P_{int} from autocorrelations following steps similar to those described above for cross-correlations.

As P_{int} modifies both the correlation functions and the related FCS generating function, it also modifies the SEE. Indeed, with interaction involved, three contributions of the non-interacting FCS generating function Eq. (S15) change to

$$
\begin{aligned}
&1 - \mathcal{T}\mathcal{T}_A(1-\mathcal{T}_B) - \mathcal{T}\mathcal{T}_B(1-\mathcal{T}_A) \to 1 - \mathcal{T}f_A(1-f_B) - \mathcal{T}f_B(1-f_A), \\
&- f_A f_B \mathcal{T}(1-\mathcal{T})(P_{\text{QPC}} + P_{\text{frac}}) \\
&\mathcal{T}\mathcal{T}_A(1-\mathcal{T}_B) \to \mathcal{T}f_A(1-f_B) + \frac{1}{2} f_A f_B \mathcal{T}(1-\mathcal{T})(P_{\text{QPC}} + P_{\text{frac}}), \\
&\mathcal{T}\mathcal{T}_B(1-\mathcal{T}_A) \to \mathcal{T}f_B(1-f_A) + \frac{1}{2} f_A f_B \mathcal{T}(1-\mathcal{T})(P_{\text{QPC}} + P_{\text{frac}}).
\end{aligned}
\tag{S57}
$$

This includes two modifications: (i) the modification of the distribution function due to fractionalization, and (ii) the interaction-induced extra bunching. The first of them can be obtained from the correction to the vertex correlation Eq. (S47). The second can be incorporated via introducing effective distribution functions. Indeed, under the influence of P_{QPC} and P_{frac}, the real distribution functions f_A and f_B in front of the central QPC behave effectively as $\tilde{f}_A$ and

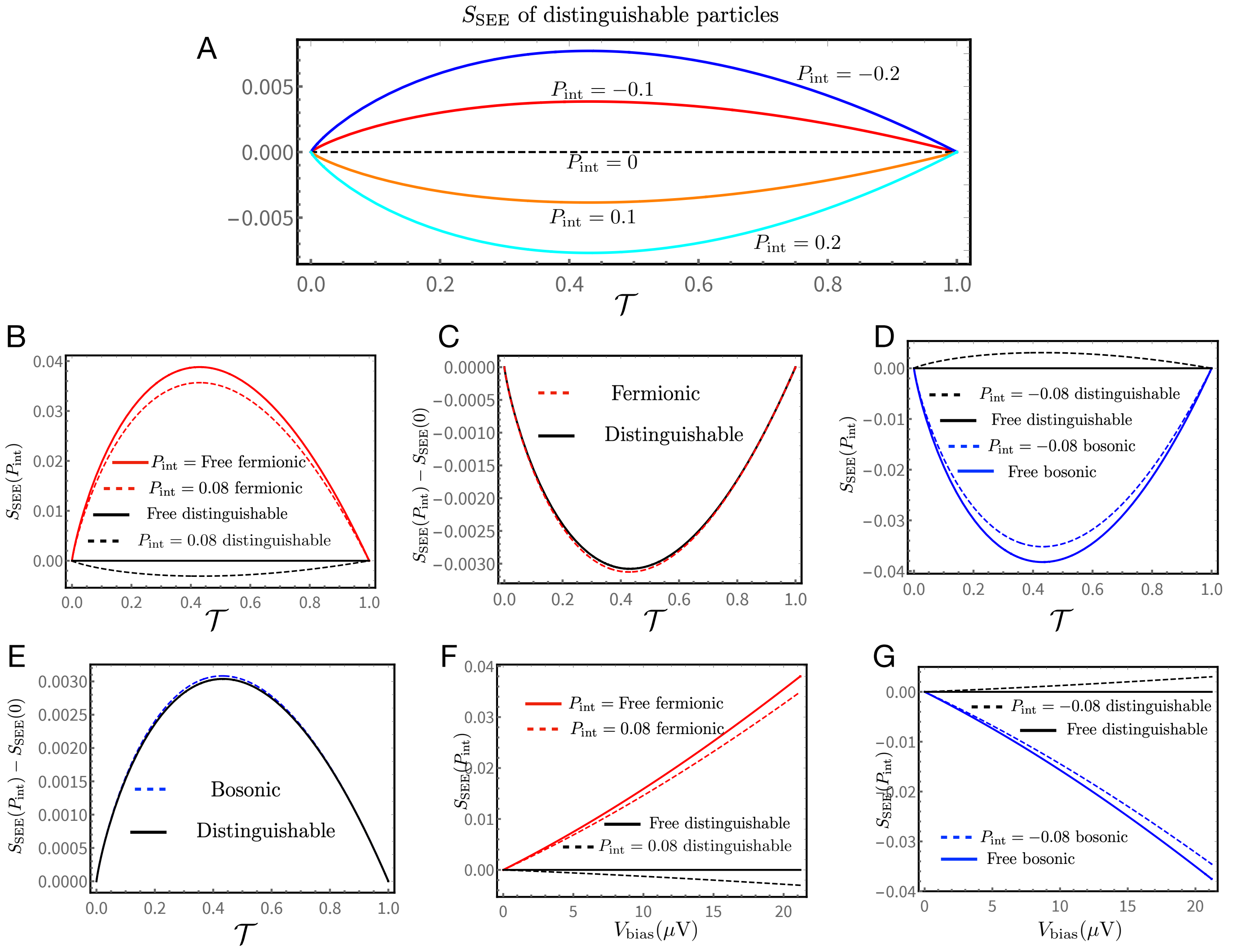

FIG. S6. Statistics-induced entanglement entropy (SEE) in the presence of interaction for $\mathcal{T}_A = \mathcal{T}_B = 0.1$. Interaction is incorporated by the parameter $P_{\rm int} = P_{\rm QPC} + P_{\rm frac}$ describing the interaction-induced bunching/anti-bunching probability. (A) The SEE of distinguishable particles with different values of $P_{\rm int}$. The dashed black line indicates the vanishing SEE for free distinguishable particles. (B) The SEE of fermions (red lines) and distinguishable particles (black lines). (C) The interaction-induced SEE of fermionic and distinguishable situations with $P_{\rm int} = 0.08$. The interaction-induced SEE is defined as the difference between dashed and solid lines with the same color, i.e., $S_{\rm SEE}(P_{\rm int}) - S_{\rm SEE}(0)$, for the dwell time $\tau = 0.01$ns, and the bias $V_{\rm bias} = 20.7\mu$V. For the given value of $P_{\rm int}$, the interaction-induced SEE is almost statistics-insensitive. (D) The SEE of bosons (blue lines) and distinguishable particles (black lines). (E) The interaction-induced SEE of bosons and distinguishable situations, with $P_{\rm int} = -0.08$. (F) and (G) show the corresponding data in (B) and (D), respectively, as a function of $V_{\rm bias}$. Here we have taken $\mathcal{T} = 0.5$ for the transmission through the central quantum point contact.

$\tilde{f}_B$. More specifically, one can obtain $\tilde{f}_A$ and $\tilde{f}_B$ by solving

$$\begin{aligned}
&\mathcal{T} f_A(1-f_B) + \frac{1}{2} f_A f_B \mathcal{T}(1-\mathcal{T})(P_{\rm QPC}+P_{\rm frac}) = \mathcal{T}\tilde{f}_A(1-\tilde{f}_B),\\
&\mathcal{T} f_B(1-f_A) + \frac{1}{2} f_A f_B \mathcal{T}(1-\mathcal{T})(P_{\rm QPC}+P_{\rm frac}) = \mathcal{T}\tilde{f}_B(1-\tilde{f}_A).
\end{aligned} \tag{S58}$$

For weak interactions, when $P_{\text{QPC}} + P_{\text{frac}}$ is a small parameter, the modification of the distribution functions approximately becomes

$$\tilde{f}_A - f_A = \tilde{f}_B - f_B \approx -\frac{f_A f_B}{2(1 - f_A - f_B)}(P_{\text{QPC}} + P_{\text{frac}})(1 - \mathcal{T}). \tag{S59}$$

With the effective distribution functions, we can write the SEE of Eq. (S21) as a sum of two terms: $S_{\text{SEE}} = S^0_{\text{SEE}} + \delta S_{\text{SEE}}$, where S^0_{SEE} follows the structure of Eq. (S21), after replacing $\mathcal{T}_A$ and $\mathcal{T}_B$ by f_A and f_B. The other part δS_{SEE}, which is generated by extra bunching at the central QPC, equals

$$\begin{aligned}\delta S_{\text{SEE}} = -\frac{e\tau}{h}\int d\epsilon \sum_{i=1,2} \{&\ln[2\cosh u_i(f_A, f_B)] - u_i(f_A, f_B)\tanh[u_i(f_A, f_B)] \\ &- \ln[2\cosh u_i(\tilde{f}_A, \tilde{f}_B)] + u_i(\tilde{f}_A, \tilde{f}_B)\tanh[u_i(\tilde{f}_A, \tilde{f}_B)]\Big\}.\end{aligned} \tag{S60}$$

Following Eq. (S59), δS_{SEE} becomes negligible for a small value of $P_{\text{int}} = P_{\text{QPC}} + P_{\text{frac}}$, where $f_A - \tilde{f}_A$ and $f_B - \tilde{f}_B$ are negligible quantities.

The SEE influenced by interaction at the central QPC is plotted in Fig. S6. In agreement with the analysis above, the correction to SEE from interactions is much smaller than the SEE of free particles, as long as $P_{\text{int}} = P_{\text{QPC}} + P_{\text{frac}}$ is a small quantity. This is, fortunately, the case of our work, where $P_{\text{int}} \approx 0.08$ as has been discussed above.

S3. STATISTICS-INDUCED ENTANGLEMENT IN TERMS OF BELL PAIRS

In this section, we show that the correlation function measurement is related to the notions of Bell pairs and Bell's inequality. We consider only the non-interacting fermionic situation in this section. We begin with the derivation of the two-fermion density matrix and the corresponding EE. We start with a two-fermion event with the initial wavefunction

$$\Psi = |\mathcal{S}_A\rangle|\mathcal{S}_B\rangle \tag{S61}$$

representing a pure state in the second quantization. This function means that one particle appears at the up source and one particle simultaneously appears at the down source. After scattering at two diluters and the central QPC, the initial wavefunction Eq. (S61) becomes

$$\begin{aligned}|\Psi\rangle = {}& r_A r_B|\tilde{\mathcal{D}}_A\rangle|\tilde{\mathcal{D}}_B\rangle + r_A t_B r|\tilde{\mathcal{D}}_A\rangle|\mathcal{D}_B\rangle - r_A t_B t|\tilde{\mathcal{D}}_A, \mathcal{D}_A\rangle|\Omega\rangle \\ &+ t_A r_B r|\mathcal{D}_A\rangle|\tilde{\mathcal{D}}_B\rangle + t_A r_B t|\Omega\rangle|\mathcal{D}_B, \tilde{\mathcal{D}}_B\rangle + t_A t_B|\mathcal{D}_A\rangle|\mathcal{D}_B\rangle,\end{aligned} \tag{S62}$$

where each term of Eq. (S62) is a product of corresponding states in subsystems A and B, respectively. For instance $|\tilde{\mathcal{D}}_A\mathcal{D}_A\rangle|\Omega\rangle$ means that both fermions end up in subsystem A ($|\tilde{\mathcal{D}}_A\mathcal{D}_A\rangle$), leaving subsystem B empty ($|\Omega\rangle$). t_A, t_B, r_A and r_B refer to the scattering matrix elements at two diluters, while t and r indicate these of the central QPC. For simplicity all these elements are assumed to be real.

After partial trace over states of subsystem B, the reduced density matrix becomes

$$\begin{aligned}\rho_A =& \mathcal{R}_A\mathcal{T}_B\mathcal{T}|\tilde{\mathcal{D}}_A,\mathcal{D}_A\rangle\langle\tilde{\mathcal{D}}_A,\mathcal{D}_A| + \mathcal{T}_A\mathcal{R}_B\mathcal{T}|\Omega\rangle\langle\Omega| \\ &+ \mathcal{R}_A(\mathcal{R}_B+\mathcal{T}_B\mathcal{R})|\tilde{\mathcal{D}}_A\rangle\langle\tilde{\mathcal{D}}_A| + \mathcal{T}_A(\mathcal{T}_B+\mathcal{R}_B\mathcal{R})|\mathcal{D}_A\rangle\langle\mathcal{D}_A| \\ &+ t_A^* r_A r|\tilde{\mathcal{D}}_A\rangle\langle\mathcal{D}_A| + t_A r_A^* r^*|\mathcal{D}_A\rangle\langle\tilde{\mathcal{D}}_A|,\end{aligned} \tag{S63}$$

where all states in Eq. (S63) belong to subsystem A. Following the definition of the von Neumann entropy, the entanglement entropy is given by

$$S_{\text{ent}} = -\sum_i \rho_i \ln \rho_i,$$

where

$$\begin{aligned}\rho_1 &= (1-\mathcal{T}_A)\mathcal{T}_B\mathcal{T}, \quad \rho_2 = \mathcal{T}_A(1-\mathcal{T}_B)\mathcal{T}, \\ \rho_3 &= \frac{1}{2}\left[1-(1-\mathcal{T}_A)\mathcal{T}_B\mathcal{T} - \mathcal{T}_A(1-\mathcal{T}_B)\mathcal{T} + \zeta_1\right], \\ \rho_4 &= \frac{1}{2}\left[1-(1-\mathcal{T}_A)\mathcal{T}_B\mathcal{T} - \mathcal{T}_A(1-\mathcal{T}_B)\mathcal{T} - \zeta_1\right]\end{aligned} \tag{S64}$$

are four eigenvalues of the reduced density matrix (S63) and

$$\zeta_1 = \sqrt{[1-\mathcal{T}(\mathcal{T}_A+\mathcal{T}_B)]^2 + 4\mathcal{T}(1-\mathcal{T})\mathcal{T}_A\mathcal{T}_B}. \tag{S65}$$

The first two eigenvalues ρ_1 and ρ_2 in Eq. (S64) correspond, respectively, to the states $|\Omega\rangle$, where both particles end in subsystem B, and $|\tilde{\mathcal{D}}_A,\mathcal{D}_A\rangle$, where they instead both stay in the subsystem A. The other two eigenvalues, ρ_3 and ρ_4, refer to two distinct configurations where each subsystem hosts a single particle. The fact that there are two non-zero eigenvalues indicates an entanglement between subsystems. Indeed, even if we know there is one state in subsystem B, the status of that in subsystem A can not be fully determined. To see the entanglement more clearly, we can rewrite the system state Eq. (S62) as

$$\begin{aligned}|\Psi\rangle =& -r_A t_B t|\tilde{\mathcal{D}}_A,\mathcal{D}_A\rangle|\Omega\rangle + t_A r_B t|\Omega\rangle|\mathcal{D}_B,\tilde{\mathcal{D}}_B\rangle \\ &+ \alpha|\uparrow_A\rangle|\uparrow_B\rangle + \beta|\downarrow_A\rangle|\downarrow_B\rangle,\end{aligned} \tag{S66}$$

where the first and second lines above refer to wavefunctions $|\tilde{\psi}\rangle$ [corresponding to configurations $(2,0)$ and $(0,2)$] and $|\psi\rangle$ [corresponding to configurations $(1,1)$], respectively.

Comparison with NOON states. In state $|\tilde{\psi}\rangle = |\psi_{2,0}\rangle + |\psi_{0,2}\rangle$, the two particles are in either one of two subsystems, resembling a NOON state (i.e., an entangled state $\sim |N\rangle|0\rangle + |0\rangle|N\rangle$, with N the particle number of the corresponding subsystem). Akin to the NOON state, the state $|\tilde{\psi}\rangle$ alone cannot directly prove the violation of Bell inequality: with only a NOON state, rotation of quasi-spin (with the z-direction of spin defined by the particle number) is impossible. Indeed, for a NOON state, the test of Bell inequality requires the fusion of the system with other external states [S13, S14]. Similarly, for our Pauli-blocked state $|\tilde{\psi}\rangle$, quantum manipulations within the subsystem are not effective; hence,

in order to probe Bell's inequality with $|\tilde{\psi}\rangle$, one needs to modify the device by coupling its arms to some external channels.

Comparison with Bell states. As discussed in the main text, the wave function $|\psi\rangle = \alpha|\uparrow_A\rangle|\uparrow_B\rangle + \beta|\downarrow_A\rangle|\downarrow_B\rangle$ is the wave function of an effective Bell pair, with orthonormal pseudospin states $|\uparrow_A\rangle, |\downarrow_A\rangle, |\uparrow_B\rangle$ and $|\downarrow_B\rangle$ [see Eq. (S67) below for their definitions]. However, $|\psi\rangle$ is not maximally entangled, since the coefficients $|\alpha|^2 = \rho_3$ and $|\beta|^2 = \rho_4$ are not, in general, equal to each other. The pseudospin-up and pseudospin-down states are given by

$$\begin{aligned}
|\uparrow_A\rangle &= \frac{2}{\zeta_A}|\tilde{\mathcal{D}}_A\rangle + \frac{-1+2\mathcal{T}_A+\mathcal{T}(\mathcal{T}_B-\mathcal{T}_A)+\zeta_1}{t_A r_A r \zeta_A}|\mathcal{D}_A\rangle \\
|\downarrow_A\rangle &= \frac{2}{\zeta_A'}|\tilde{\mathcal{D}}_A\rangle + \frac{-1+2\mathcal{T}_A+\mathcal{T}(\mathcal{T}_B-\mathcal{T}_A)-\zeta_1}{t_A r_A r \zeta_A'}|\mathcal{D}_A\rangle \\
|\uparrow_B\rangle &= \frac{2}{\zeta_B}|\tilde{\mathcal{D}}_B\rangle + \frac{-1+2\mathcal{T}_B+\mathcal{T}(\mathcal{T}_A-\mathcal{T}_B)+\zeta_1}{t_B r_B r \zeta_B}|\mathcal{D}_B\rangle \\
|\downarrow_B\rangle &= \frac{2}{\zeta_B'}|\tilde{\mathcal{D}}_B\rangle + \frac{-1+2\mathcal{T}_B+\mathcal{T}(\mathcal{T}_A-\mathcal{T}_B)-\zeta_1}{t_B r_B r \zeta_B'}|\mathcal{D}_B\rangle,
\end{aligned} \tag{S67}$$

where

$$\begin{aligned}
\zeta_A &= \sqrt{4+\frac{[-1+2\mathcal{T}_A+\mathcal{T}(\mathcal{T}_B-\mathcal{T}_A)+\zeta_1]^2}{\mathcal{T}_B(1-\mathcal{T}_B)(1-\mathcal{T})}}, \quad \zeta_A' = \sqrt{4+\frac{[-1+2\mathcal{T}_A+\mathcal{T}(\mathcal{T}_B-\mathcal{T}_A)-\zeta_1]^2}{\mathcal{T}_B(1-\mathcal{T}_B)(1-\mathcal{T})}}, \\
\zeta_B &= \sqrt{4+\frac{[-1+2\mathcal{T}_B-\mathcal{T}(\mathcal{T}_B-\mathcal{T}_A)+\zeta_1]^2}{\mathcal{T}_A(1-\mathcal{T}_A)(1-\mathcal{T})}}, \quad \zeta_B' = \sqrt{4+\frac{[-1+2\mathcal{T}_B-\mathcal{T}(\mathcal{T}_B-\mathcal{T}_A)-\zeta_1]^2}{\mathcal{T}_A(1-\mathcal{T}_A)(1-\mathcal{T})}}
\end{aligned} \tag{S68}$$

are normalization factors. When $\mathcal{T}_A = \mathcal{T}_B$, these functions can be greatly simplified, leading to

$$\begin{aligned}
|\uparrow_A\rangle &= \frac{2}{\zeta_A}|\tilde{\mathcal{D}}_A\rangle + \frac{-1+2\mathcal{T}_A+\zeta_1}{t_A r_A r \zeta_A}|\mathcal{D}_A\rangle \\
|\downarrow_A\rangle &= \frac{2}{\zeta_A'}|\tilde{\mathcal{D}}_A\rangle + \frac{-1+2\mathcal{T}_A-\zeta_1}{t_A r_A r \zeta_A'}|\mathcal{D}_A\rangle \\
|\uparrow_B\rangle &= \frac{2}{\zeta_B}|\tilde{\mathcal{D}}_B\rangle + \frac{-1+2\mathcal{T}_A+\zeta_1}{t_A r_A r \zeta_B}|\mathcal{D}_B\rangle \\
|\downarrow_B\rangle &= \frac{2}{\zeta_B'}|\tilde{\mathcal{D}}_B\rangle + \frac{-1+2\mathcal{T}_A-\zeta_1}{t_A r_A r \zeta_B'}|\mathcal{D}_B\rangle,
\end{aligned} \tag{S69}$$

where $\zeta_1 = \sqrt{1-4\mathcal{T}(1-\mathcal{T}_A)\mathcal{T}_A}$, and

$$\begin{aligned}
\zeta_A = \zeta_B &= \sqrt{4+\frac{[-1+2\mathcal{T}_A+\sqrt{1-4\mathcal{T}(1-\mathcal{T}_A)\mathcal{T}_A}]^2}{(1-\mathcal{T})(\mathcal{T}_A-1)\mathcal{T}_A}} \\
\zeta_A' = \zeta_B' &= \sqrt{4-\frac{[-1+2\mathcal{T}_A-\sqrt{1-4\mathcal{T}(1-\mathcal{T}_A)\mathcal{T}_A}]^2}{(1-\mathcal{T})(\mathcal{T}_A-1)\mathcal{T}_A}}
\end{aligned} \tag{S70}$$

Now we consider the connection of Eq. (S67) to the Bell pairs. In experiments, one measures the correlation $\langle I_A I_B\rangle$, which records the simultaneous appearance of particles in $\mathcal{D}_A$ and $\mathcal{D}_B$. If we limit ourselves to configurations where each subsystem hosts a single particle of a two-particle state, we can introduce spin in each subsystem. Indeed, in these situations, we can consider subsystem A as spin-up along z-axis if the particle in A (B) is in state $|\uparrow_A\rangle$ ($|\uparrow_B\rangle$), and spin-down in state $|\downarrow_A\rangle$ ($|\downarrow_B\rangle$). In this sense, the current measurement $\langle I_A I_B\rangle$ can be considered as a

spin measurement along some directions that deviate from the z-axis. It is then related to the measurement of the Bell inequality, similar to that proposed by Ref. [S15].

S4. ENTANGLEMENT ENTROPY FROM SINGLE-PARTICLE AND TWO-PARTICLE SCATTERING PICTURES

In the previous section, we have shown that for a two-particle scattering event, the outcome can be categorized into two types of configurations: (i) states $|\tilde{\psi}\rangle$, with particles in the same subsystem [i.e., $(2,0)$ and $(0,2)$], and (ii) states $|\psi\rangle$, where each subsystem hosts one particle [i.e., configurations of (1,1)]. In this section, we look into both EE and corresponding SEE contributions from these two types of configurations.

Briefly, following Sec. S3, after scattering events at three QPCs, the entanglement equals $S_{\text{ent}} = -\sum_i \rho_i \ln \rho_i$, with ρ_i [given by Eq. (S64)] eigenvalues of the reduced density matrix ρ_A. Here ρ_1, ρ_2 correspond to configurations $(2,0)$ and $(0,2)$, respectively. The other two eigenvalues ρ_3, ρ_4 correspond to configurations of the $(1,1)$ type. It is thus straightforward to define EE from $|\tilde{\psi}\rangle$ as $S^{(2,0)} + S^{(0,2)} \equiv -\rho_1 \ln \rho_1 - \rho_2 \ln \rho_2$, and that from $|\psi\rangle$ as $S^{(1,1)} \equiv -\rho_3 \ln \rho_3 - \rho_4 \ln \rho_4$. We can further define SEE of $|\tilde{\psi}\rangle$ and $|\psi\rangle$, following Eq. (4) of the main text, as

$$S_{\text{SEE}}^{(N_A,N_B)} \equiv S^{(N_A,N_B)}(0,\mathcal{T}_B) + S^{(N_A,N_B)}(\mathcal{T}_A,0) - S^{(N_A,N_B)}(\mathcal{T}_A,\mathcal{T}_B), \tag{S71}$$

for the configuration (N_A, N_B) of the pure two-particle ($N_A + N_B = 2$) state with N_A particle in subsystem A and N_B particles in subsystem B. By doing so, we are removing statistics-irrelevant configurations in Fig. S7, where two involved particles scatter independently. The obtained EE and SEE are provided in Table I.

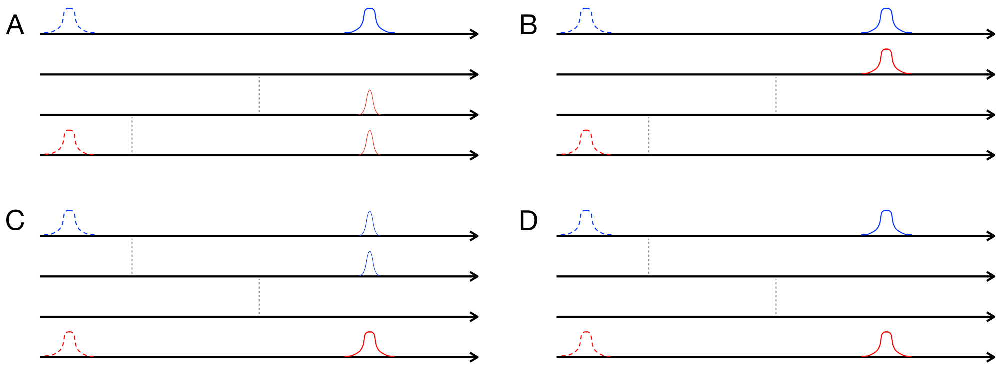

FIG. S7. Configurations whose entanglement entropy contributions are removed from statistics-induced entanglement entropy. Two initial states of the particles are indicated by dashed pulses. Panels A and B show the final states, for configurations $(1,1)$ and $(2,0)$, respectively, when $\mathcal{T}_A = 0$. Panel C and D present the corresponding configurations when $\mathcal{T}_B = 0$.

As shown in Table I, for particles with fermionic statistics, both $|\tilde{\psi}\rangle$ [corresponding to $(2,0)$ and $(0,2)$ configurations] and $|\psi\rangle$ [corresponding to $(1,1)$ configurations] have finite contributions to SEE. In the strongly diluted limit,

	Configurations $(2,0)$ and $(0,2)$	Configuration $(1,1)$
EE	$S^{(2,0)}+S^{(0,2)}=-\rho_1\ln\rho_1-\rho_2\ln\rho_2$	$S^{(1,1)}=-\rho_3\ln\rho_3-\rho_4\ln\rho_4$
Leading-order SEE	$S_{\text{SEE}}^{(2,0)}+S_{\text{SEE}}^{(0,2)}$ $\approx -\mathcal{T}\mathcal{T}_A\mathcal{T}_B[2+\ln(\mathcal{T}^2\mathcal{T}_A\mathcal{T}_B)]$	$S_{\text{SEE}}^{(1,1)}$ $\approx \mathcal{T}\mathcal{T}_A\mathcal{T}_B[2+\mathcal{T}\ln(\mathcal{T}_A\mathcal{T}_B\mathcal{T}^2)]$

TABLE I. Contributions of configurations $(N_A=2,N_B=0),(N_A=0,N_B=2)$ and $(N_A=1,N_B=1)$ to the EE and SEE of fermions. SEE is defined following Eq. (4) of the main text, i.e., $S_{\text{SEE}}^{(N_A,N_B)}=S^{(N_A,N_B)}(0,\mathcal{T}_B)+S^{(N_A,N_B)}(\mathcal{T}_A,0)-S^{(N_A,N_B)}(\mathcal{T}_A,\mathcal{T}_B)$, for the configuration (N_A,N_B).

their sum equals to $S_{\text{SEE}}=S_{\text{SEE}}^{(2,0)}+S_{\text{SEE}}^{(0,2)}+S_{\text{SEE}}^{(1,1)}\approx-\mathcal{T}(1-\mathcal{T})\mathcal{T}_A\mathcal{T}_B\ln(\mathcal{T}^2\mathcal{T}_A\mathcal{T}_B)$, in agreement with Eq. (11) of the main text. In addition, in the strongly diluted limit, the ratio of the two contributions to the SEE reads as

$$\frac{S_{\text{SEE}}^{(1,1)}}{S_{\text{SEE}}^{(2,0)}+S_{\text{SEE}}^{(0,2)}}=-\frac{2+\mathcal{T}\ln(\mathcal{T}^2\mathcal{T}_A\mathcal{T}_B)}{2+\ln(\mathcal{T}^2\mathcal{T}_A\mathcal{T}_B)}. \tag{S72}$$

Equation (S72) demonstrates that, generically, both $S_{\text{SEE}}^{(1,1)}$ and $S_{\text{SEE}}^{(2,0)}+S_{\text{SEE}}^{(0,2)}$ have a finite contribution to SEE.

Before closing this section, we emphasize that, although SEE contains contributions from both $|\tilde{\psi}\rangle$ and $|\psi\rangle$, it does not influence the correlation between Bell nonlocality and a finite SEE. Indeed, if SEE equals zero, two contributing particles become distinguishable from each other. In this case, the two-particle scattered wave function does not contain $|\psi\rangle$, and is thus unable to violate Bell inequality.

S5. THE DWELL TIME AND THE MEASURING TIME

In general, we can assume that a certain type of scattering event i produces the time-independent EE $s_{i,A}$ between two subsystems. Here i can be either a single-particle or multi-particle scattering event. Assuming such an event occurs N_i times during the entire measurement, a total EE $S_{\text{ent}}=\sum_i N_i s_{i,A}$ is then produced.

In systems with constant sources, $N_i\propto\tau_{\text{measure}}$ for each type of scattering, where τ_{measure} is the measuring time (of FCS) during which two subsystems become entangled. Theoretically $S_{\text{ent}}\propto\tau_{\text{measure}}$ thus also grows linearly in the measuring time. This time-dependence is indeed naturally expected, as we are evaluating the EE from the FCS generating function that is known to be proportional to τ_{measure}. However, in experiments, EE cannot grow indefinitely in time, as particles lose coherence after entering the drains. As a consequence, experimentally S_{ent} (and also S_{SEE}) becomes instead proportional to the dwell time τ, which is the time needed for a particle to travel from the central QPC to one of the drains.

In this work, t_{dwell} can be considered as the traveling time from the central QPC to either drain. More specifically, it is given by the ratio of the distance between the central QPC and the closest drain (around 5μm) and the traveling velocity (around 4.6×10^5m/s), which yields $t_{\text{dwell}}\approx0.01$ns.

S6. ADDITIONAL EXPERIMENTAL DATA

S6A. Experimental data of the $\nu = 3$ integer quantum Hall sample

In Sec. S2, we have analyzed correlation functions in the presence of interaction, and have compared the theoretical results with the measured data. In this section, we present more data in Fig. S8.

As illustrated in the main text, the error bars stand for standard deviation of the mean for the set of measurements. Actually, since the number of measurements is finite, the error bars equivalently represent for the 1σ standard deviations of the sample mean (SDSM) of transmissions (left panel of Fig. S8) and cross-correlations (right panel of Fig. S8). More specifically, for a quantity Q (transmission or cross-correlation), its SDSM, obtained with measurement, is defined as

$$\text{SDSM}(Q) \equiv \frac{\sqrt{\sum_{i=1}^{N}(Q_i - \bar{Q})^2}}{N}, \tag{S73}$$

where Q_i is the outcome of the ith measurement, and $\bar{Q}$ is the sample mean $\bar{Q} \equiv \sum_{i=1}^{N} Q_i/N$, after N measurements. In practical experiments, N is a finite number, and the obtained sample mean $\bar{Q}$ fluctuates around the real mean value (which, ideally, is obtained after infinite times of measurements). The 1σ standard deviation of this fluctuation equals SDSM defined in Eq. (S73).

For single-source cases, all transmissions are measured for four times. For double-source cases, we instead measure $\mathcal{T}_A$ and $\mathcal{T}_B$ for three times, and $\mathcal{T}_{\text{Center}}$ for eight times. As for cross-correlation measurements, single-source noises are measured 442 and 565 times, respectively, for cases with either $\mathcal{S}_A$ or $\mathcal{S}_B$ on. They are measured 461 times with both sources on.

S6B. Correlations for filling factor $\nu = 1$: emergence of non-linearity

In this section, we present the measured correlation functions in an IQH sample with filling factor 1. The results are shown in Fig. S9. Surprisingly, the results display strong non-linearity. Indeed, in Figs. S9B, C, and D, transmission probabilities at all three QPCs become current-bias dependent, indicating the possible presence of interaction. Accompanying the bias-dependence of transmissions, we also observe a negative double-source cross-correlation $\langle I_A I_B\rangle - \langle I_A\rangle\langle I_B\rangle$, as displayed in Fig. S9D. We anticipate that this negative cross-correlation reflects a modified bunching probability, i.e., a finite P_{QPC} of Eq. (S51), which becomes manifest owing to the absence of charge fractionalization in a $\nu = 1$ system. Indeed, in a $\nu = 3$ system (see Fig. 5B), the equal-source cross-correlation is positive. Following Eq. (S51), this positive cross-correlation is expected to reflect the sign of the leading interaction-induced contributions, i.e., terms proportional to $\mathcal{T}_A P_A$ and $\mathcal{T}_B P_B$ of Eq. (S51). Importantly, these leading interaction-induced corrections require charge fractionalization between modes of multiple channels and are thus impossible for the $\nu = 1$ system. The next-order correction involving P_{QPC}, which reflects the interaction-induced modification of the bunching

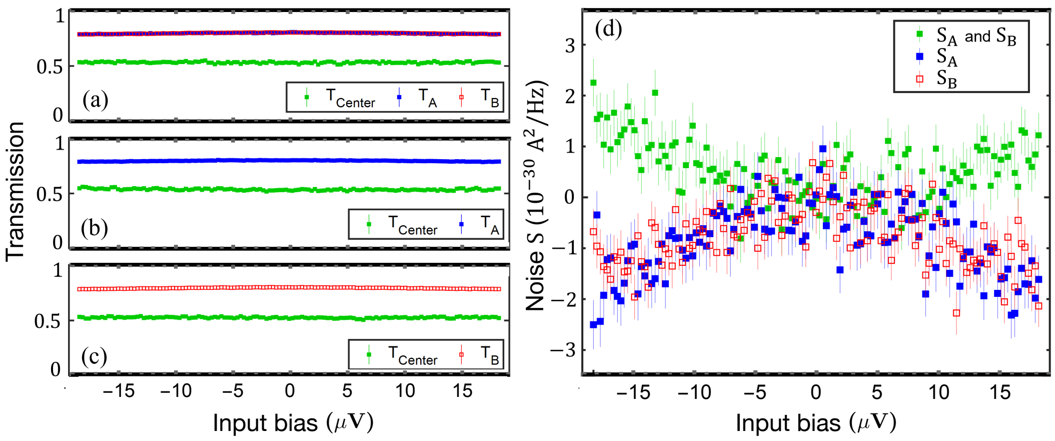

FIG. S8. Experimental data for the $\nu = 3$ sample. Here, the blue, red, and green data points (with error bars) represent the transmission probabilities at the upper diluter, lower diluter, and central quantum point contact, respectively. The error bars represent standard deviation of the sample mean of transmissions (the left panel) and cross-correlation noise (the right panel). (A) Transmission probabilities with both sources on; (B) Transmission probabilities with only the upper source (S_A) on, and (C) that with only the lower source (S_B) on. (D) Corresponding measured cross-correlations.

preference due to interactions at the central QPC, becomes then the leading one and thus is easily observable. This negative noise shown in Fig. S9D then perfectly agrees with our analysis of Eq. (S51).

S7. Possible SEE application: quantifying entanglement of a mixed state

Our proposed framework can potentially help the quantification of the entanglement of mixed states, such as those in the setup displayed in Fig. S10A, where the system $D_1 + D_2$ couples to the environment. In this situation, the formally evaluated entanglement entropy of the system comprising D_1 and D_2 does not quantify entanglement between the subsystems, but is rather dominated by the interaction with the environment. Indeed, after a naive partial trace over states in D_2, the EE contains entanglement from both interfaces γ_{inner} and γ_{outer}.

To quantify entanglement in mixed states, functions such as logarithmic entanglement negativity (see, e.g., Refs. [S18, S19]) have been discussed in the literature. In addition, the hierarchy of quantum correlations, including the EPR steering and Bell nonlocality were introduced, to classify entanglement of mixed states: entanglement $<$ EPR steering $<$ Bell nonlocality (see, e.g., Ref. [S20], and related discussions of Ref. [S21, S22]). Denoted as hierarchy, it means that Bell nonlocality of a mixed state is sufficient to perform EPR steering (and to prove entanglement), but not the other way around (i.e., not all entangled mixed state can realize EPR steering; not every EPR steerable mixed state is subject to Bell's nonlocality inequality). The entanglement indicators proposed in the present work give an alternative idea for obtaining the entanglement of mixed states. Indeed, the redundant entropy from system-environment communication is naturally excluded following our proposed functions (EP and SEE), similarly to the

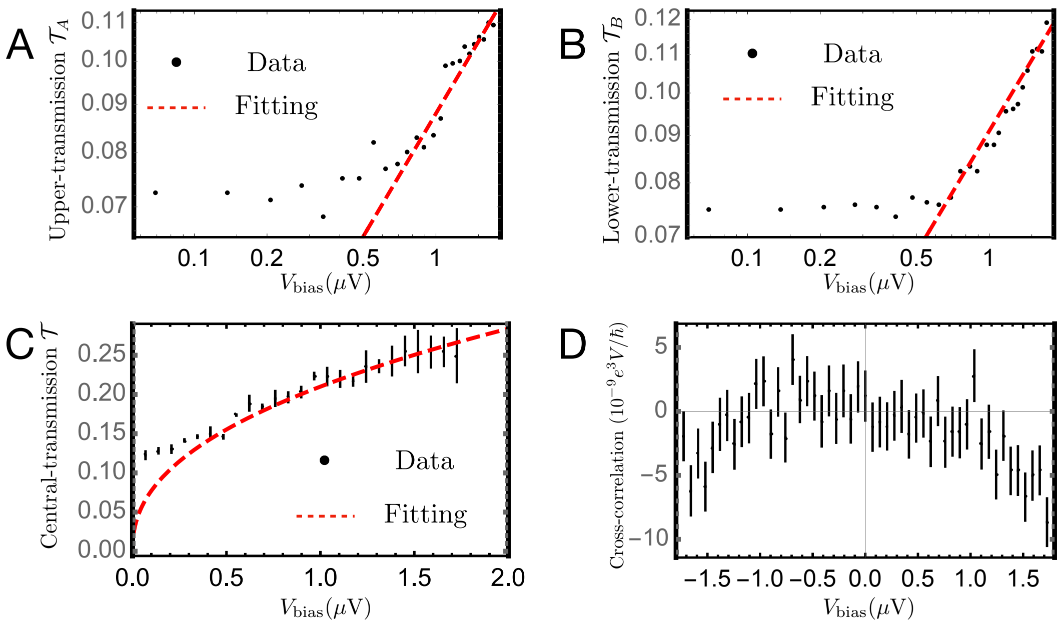

FIG. S9. Experimental data for the $\nu = 1$ sample. The error bars represent standard deviation of the sample mean, of measured quantities. Panels (A), (B), and (C) show the transmission probabilities at three quantum point contacts (QPCs) as functions of the bias voltage. At high bias, the transmissions of all three QPCs can be approximately fitted by a power-law function of the non-equilibrium current $\propto I^{0.435}$ (red dashed) line. This behavior can originate from either Luttinger-liquid renormalization (see, e.g., Ref. [S16]) resulting from the interactions between particles in counter-propagating channels, or from excitation emerging from edge reconstruction. Panel (D) shows the irreducible cross-correlation $\langle I_A I_B\rangle - \langle I_A\rangle\langle I_B\rangle$ as a function of the current bias, when two sources are equally diluted. In contrast to the $\nu = 3$ results of Fig. 5C, the double-source cross-correlation with $\nu = 1$ is negative.

cancellation of such contributions in mutual information. This is particularly true if the two involved subsystems become entangled through exchanging anyons or chiral Majorana modes (Fig. S10B), as they are normally not involved in the system-environment entanglement.

S8. EE and SEE with a finite scattering area

In our work, we extract the information on entanglement, i.e., the EE and SEE between two subsystems of an HOM interferometer, from current correlations. In our method, the obtained SEE reflects entanglement induced by particles after the central QPC. In general, when scattering between subsystems occurs at multiple positions (Fig. S11), one also has to consider the EE of particles in the scattering regime.

To see this point more explicitly, we start with the simple situation of Fig. S11A. We consider the EE produced by a single particle from the right part, with the system described by the initial wavefunction $|R\rangle$. This setup is equivalent to a Mach-Zehnder interferometer. After scattering at both QPCs, the final wavefunction can be written as

$$(t_1 r_2 + r_1 t_2)|L\rangle + (-t_1 t_2 + r_1 r_2)|R\rangle, \tag{S74}$$

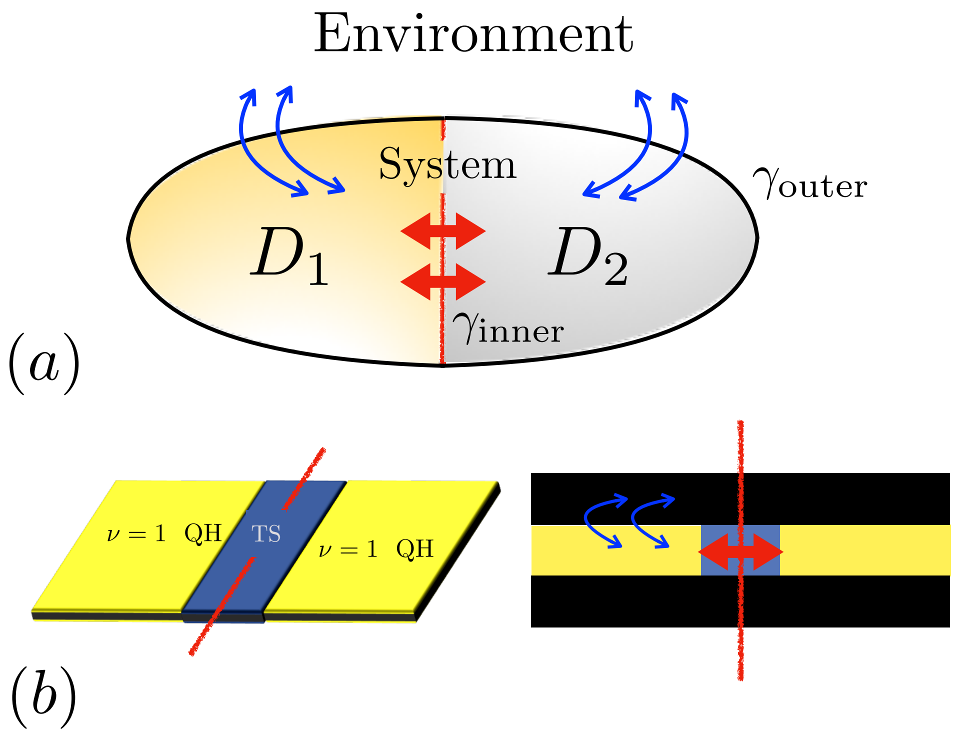

FIG. S10. Another possible application of our work: quantifying entanglement of mixed states. (A) A mixed state where the system comprising subsystems D_1 and D_2 interacts with its environment. After the partial trace over states in D_2, the calculated EE involves contributions from both interfaces γ_{inner} and γ_{outer}. The communication between the system and the environment and the D_1–D_2 communications involve different types of quasiparticles, indicated by different types of arrows. (B) An example to illustrate our point. Left panel: the system that contains Majorana chiral modes (marked by the black arrow) at the interface of a $\nu = 1$ quantum Hall (QH) sample and a topological superconductor (TS) [S17]. Right panel: experimentally, the entanglement entropy involves contributions from both chiral Majorana modes (the thick red double arrow) and the system-environment communication with fermions (the blue double arrows). The latter entanglement contribution can be excluded in case one focuses on only statistics-induced entanglement entropy from chiral Majorana modes.

where t_1, r_1, t_2, and r_2 are the scattering matrix parameters for the first and second QPCs, respectively. Consider, for instance, the case of destructive interference $t_1 r_2 = -r_1 t_2$. In this case, when the particle has traveled after the second QPC, it always stays in the previous (right) subsystem, leading to a vanishing EE. However, one cannot conclude that these two subsystems never become entangled. Indeed, when the particle is between the two QPCs, it introduces finite EE $\mathcal{T}_1 \ln \mathcal{T}_1 + (1 - \mathcal{T}_1) \ln(1 - \mathcal{T}_1)$, where $\mathcal{T}_1 \equiv t_1^2$ is the transmission probability of the first QPC.

Now, if we go to the more general case presented in Fig. S11B, the EE and SEE contain two contributions: from particles after the scattering area and from those within the scattering area. The former contribution can be figured out by obtaining the effective transmission probability $\mathcal{T}_{\text{sa}}$ of the entire scattering area. Indeed, one can obtain EE of the former contribution by substituting transmission $\mathcal{T}$ of the HOM geometry by $\mathcal{T}_{\text{sa}}$. This contribution grows linearly with the measurement time, as long as this time is smaller than other possible cutoffs in time, e.g., the dephasing time.

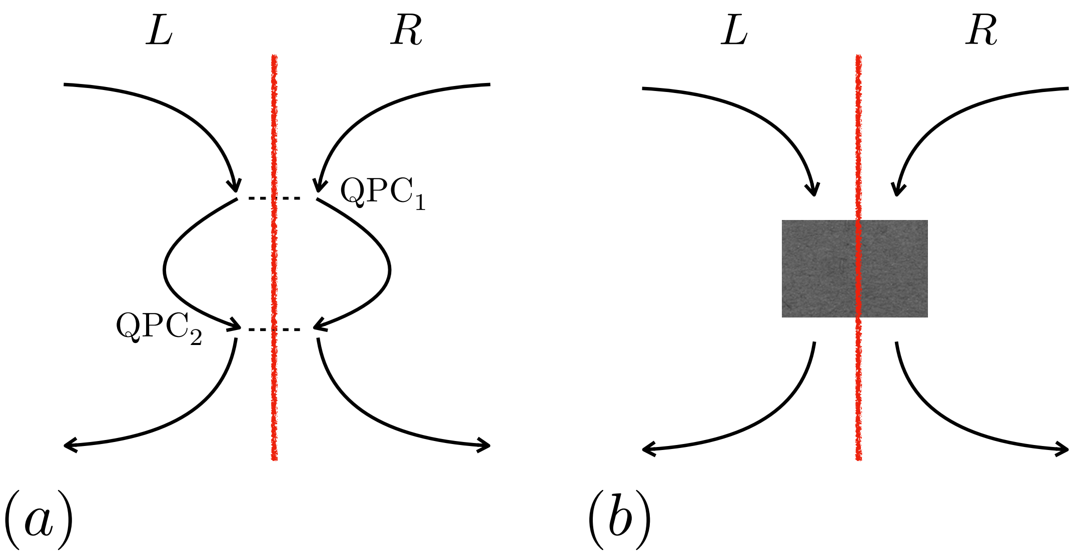

FIG. S11. Situations with (A) two scattering quantum point contacts and (B) a scattering area (the grey box). Other required elements, e.g., diluters, are not shown in the figure.

In contrast, entanglement from particles within the scattering area is constant if the measurement time τ is larger than the traveling time within the QPC. Specifically, in the two-QPC structure shown in Fig. S11, the EE and SEE from particles between two QPCs can be obtained by (i) replacing $\mathcal{T}$ by $\mathcal{T}_1$ of the first QPC and (ii) replacing the measuring time τ by the traveling time between two QPCs. As a consequence, the EE and SEE from inter-QPC particles are negligible once the inter-QPC traveling time is much smaller than the dephasing time of the system.

[S1] Israel Klich and Leonid Levitov, "Quantum noise as an entanglement meter," Phys. Rev. Lett. **102**, 100502 (2009).

[S2] Israel Klich and Leonid Levitov, "Many-body entanglement: a new application of the full counting statistics," AIP Conference Proceedings **1134**, 36–45 (2009), https://aip.scitation.org/doi/pdf/10.1063/1.3149497.

[S3] H. Francis Song, Christian Flindt, Stephan Rachel, Israel Klich, and Karyn Le Hur, "Entanglement entropy from charge statistics: Exact relations for noninteracting many-body systems," Phys. Rev. B **83**, 161408 (2011).

[S4] A. O. Gogolin and A. Komnik, "Towards full counting statistics for the Anderson impurity model," Phys. Rev. B **73**, 195301 (2006).

[S5] Claire Wahl, Jérôme Rech, Thibaut Jonckheere, and Thierry Martin, "Interactions and charge fractionalization in an electronic Hong-Ou-Mandel interferometer," Phys. Rev. Lett. **112**, 046802 (2014).

[S6] C. de C. Chamon and X. G. Wen, "Sharp and smooth boundaries of quantum hall liquids," Phys. Rev. B **49**, 8227–8241 (1994).

[S7] C. L. Kane and Matthew P. A. Fisher, "Impurity scattering and transport of fractional quantum hall edge states," Phys. Rev. B **51**, 13449–13466 (1995).

[S8] I. Safi, "A dynamic scattering approach for a gated interacting wire," The European Physical Journal B - Condensed Matter

and Complex Systems **12**, 451–455 (1999).

[S9] Edvin G. Idrisov, Ivan P. Levkivskyi, Eugene V. Sukhorukov, and Thomas L. Schmidt, "Current cross-correlations in a quantum Hall collider at filling factor two," arXiv:2201.08147 , arXiv:2201.08147 (2022), arXiv:2201.08147 [cond-mat.mes-hall].

[S10] Ivan P. Levkivskyi and Eugene V. Sukhorukov, "Energy relaxation at quantum Hall edge," Phys. Rev. B **85**, 075309 (2012).

[S11] Sungguen Ryu and H. S. Sim, "Partition of Two Interacting Electrons by a Potential Barrier," arXiv:2207.01473 , arXiv:2207.01473 (2022), arXiv:2207.01473 [cond-mat.mes-hall].

[S12] L. Bellentani, P. Bordone, X. Oriols, and A. Bertoni, "Coulomb and exchange interaction effects on the exact two-electron dynamics in the Hong-Ou-Mandel interferometer based on Hall edge states," Phys. Rev. B **99**, 245415 (2019).

[S13] S. M. Tan, D. F. Walls, and M. J. Collett, "Nonlocality of a single photon," Phys. Rev. Lett. **66**, 252–255 (1991).

[S14] Björn Hessmo, Pavel Usachev, Hoshang Heydari, and Gunnar Björk, "Experimental demonstration of single photon nonlocality," Phys. Rev. Lett. **92**, 180401 (2004).

[S15] Nikolai M. Chtchelkatchev, Gianni Blatter, Gordey B. Lesovik, and Thierry Martin, "Bell inequalities and entanglement in solid-state devices," Phys. Rev. B **66**, 161320 (2002).

[S16] P. Fendley, A. W. W. Ludwig, and H. Saleur, "Exact nonequilibrium dc shot noise in Luttinger liquids and fractional quantum Hall devices," Phys. Rev. Lett. **75**, 2196–2199 (1995).

[S17] Roger S. K. Mong, David J. Clarke, Jason Alicea, Netanel H. Lindner, Paul Fendley, Chetan Nayak, Yuval Oreg, Ady Stern, Erez Berg, Kirill Shtengel, and Matthew P. A. Fisher, "Universal topological quantum computation from a superconductor-Abelian quantum Hall heterostructure," Phys. Rev. X **4**, 011036 (2014).

[S18] M. B. Plenio, "Logarithmic negativity: A full entanglement monotone that is not convex," Phys. Rev. Lett. **95**, 090503 (2005).

[S19] Ryszard Horodecki, Paweł Horodecki, Michał Horodecki, and Karol Horodecki, "Quantum entanglement," Rev. Mod. Phys. **81**, 865–942 (2009).

[S20] H. M. Wiseman, S. J. Jones, and A. C. Doherty, "Steering, entanglement, nonlocality, and the einstein-podolsky-rosen paradox," Phys. Rev. Lett. **98**, 140402 (2007).

[S21] Daniel Collins, Nicolas Gisin, Noah Linden, Serge Massar, and Sandu Popescu, "Bell inequalities for arbitrarily high-dimensional systems," Phys. Rev. Lett. **88**, 040404 (2002).

[S22] R. Y. Teh, L. Rosales-Zárate, B. Opanchuk, and M. D. Reid, "Signifying the nonlocality of noon states using einstein-podolsky-rosen steering inequalities," Phys. Rev. A **94**, 042119 (2016).
